# ABCC6, Pyrophosphate and Ectopic Calcification: Therapeutic Solutions

**DOI:** 10.3390/ijms22094555

**Published:** 2021-04-27

**Authors:** Briana K. Shimada, Viola Pomozi, Janna Zoll, Sheree Kuo, Ludovic Martin, Olivier Le Saux

**Affiliations:** 1 Department of Cell and Molecular Biology, John A. Burns School of Medicine, University of Hawaii at Manoa, Honolulu, HI 96817, USA; bkshimad@hawaii.edu (B.K.S.); jzoll@hawaii.edu (J.Z.); 2 Institute of Enzymology, RCNS, Hungarian Academy of Sciences, 1117 Budapest, Hungary; pomozi.viola@gmail.com; 3 Department of Pediatrics, Kapi’olani Medical Center for Women and Children, University of Hawaii, Honolulu, HI 96826, USA; kuos@hawaii.edu; 4 PXE Consultation Center, MAGEC Reference Center for Rare Skin Diseases, Angers University Hospital, 49100 Angers, France; LuMartin@chu-angers.fr; 5 BNMI, CNRS 6214/INSERM 1083, University Bretagne-Loire, 49100 Angers, France

**Keywords:** calcification, ABCC6, pseudoxanthoma elasticum, generalized arterial calcification of infancy, pyrophosphate, therapies

## Abstract

Pathological (ectopic) mineralization of soft tissues occurs during aging, in several common conditions such as diabetes, hypercholesterolemia, and renal failure and in certain genetic disorders. Pseudoxanthoma elasticum (PXE), a multi-organ disease affecting dermal, ocular, and cardiovascular tissues, is a model for ectopic mineralization disorders. ABCC6 dysfunction is the primary cause of PXE, but also some cases of generalized arterial calcification of infancy (GACI). ABCC6 deficiency in mice underlies an inducible dystrophic cardiac calcification phenotype (DCC). These calcification diseases are part of a spectrum of mineralization disorders that also includes Calcification of Joints and Arteries (CALJA). Since the identification of ABCC6 as the “PXE gene” and the development of several animal models (mice, rat, and zebrafish), there has been significant progress in our understanding of the molecular genetics, the clinical phenotypes, and pathogenesis of these diseases, which share similarities with more common conditions with abnormal calcification. ABCC6 facilitates the cellular efflux of ATP, which is rapidly converted into inorganic pyrophosphate (PPi) and adenosine by the ectonucleotidases NPP1 and CD73 (NT5E). PPi is a potent endogenous inhibitor of calcification, whereas adenosine indirectly contributes to calcification inhibition by suppressing the synthesis of tissue non-specific alkaline phosphatase (TNAP). At present, therapies only exist to alleviate symptoms for both PXE and GACI; however, extensive studies have resulted in several novel approaches to treating PXE and GACI. This review seeks to summarize the role of ABCC6 in ectopic calcification in PXE and other calcification disorders, and discuss therapeutic strategies targeting various proteins in the pathway (ABCC6, NPP1, and TNAP) and direct inhibition of calcification via supplementation by various compounds.

## 1. Introduction

Physiological calcification is a multifactorial metabolic process normally restricted to the bones and teeth. Calcification and mineralization (we will use these terms interchangeably thereon) primarily designate the formation of apatite crystals. Apatite is made of phosphate and calcium ions (Ca_10_(PO_4_)_6_(OH)_2_), but the crystal formation happens via a number of short-lived intermediates such as octacalcium phosphate and amorphous calcium phosphate [1]. The intra- and extracellular mechanisms regulating mineralization rest upon a tightly regulated balance between calcification inhibitors and promoters. Under normal circumstances, calcium and inorganic phosphate (Pi) concentrations are near saturation in most soft tissues, which necessitates strong calcification inhibition systems [2].

**Figure 1:**
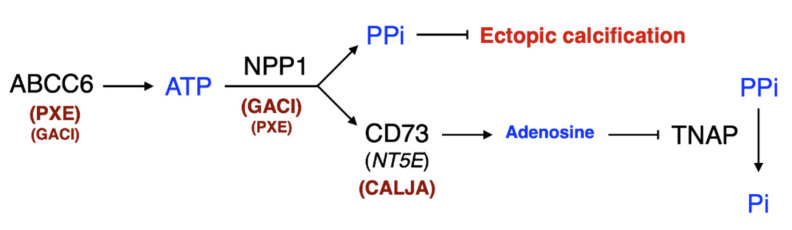
The ABCC6 pathway influences calcification and extracellular purinergic metabolism. ABCC6 facilitates the cellular efflux of ATP from liver and other tissues/cells, which is quickly converted to pyrophosphate (PPi), a potent inhibitor of mineralization. Decreased plasma PPi levels cause calcification in PXE and GACI. CD73 activity leads to adenosine production, which affects many biological activities including the inhibition of TNAP synthesis. TNAP degrades PPi into inorganic phosphate (Pi), an activator of calcification, which leads to vascular calcification in CAL JA patients.

Ectopic calcification can lead to clinical symptoms when it occurs in cardiovascular tissues and has been the object of intense research focus. Many host, environmental, and genetic factors contributing to this calcification have been identified [3], but there are still gaps in our understanding [4]. In recent years, the identification of mutations in the ATP-binding cassette (ABC) transporter ABCC6 [5,6,7] and the characterization of its function [8] has provided new molecular insight into the regulation of ectopic calcification inhibition in relation to pyrophosphate (PPi). ABCC6 mediates the cellular efflux of nucleotides, notably ATP, which is rapidly converted into PPi and adenosine at the cellular surface by the ectonucleotidases NPP1 (encoded by *ENPP1*) and CD73 (encoded by *NT5E*) [8,9,10,11]. Both PPi and indirectly adenosine are major inhibitors of calcification. ABCC6 deficiencies underlie the calcification disorders PXE (MIM#264800) and a cardiac calcification phenotype (DCC) described in mice [12,13,14]. GACI (MIM#208000) is primarily linked to NPP1, the key enzyme that generates PPi [15]. However, some cases of GACI (MIM#614473) are caused by *ABCC6* mutations, while some PXE patients carry disease-causing variants only in *ENPP1* [16,17]. CALJA (MIM#211800) is due to mutations in CD73 (*NT5E*) [10]. PXE, together with GACI and CALJA form a spectrum of diseases with overlapping calcification processes but with distinct clinical features. If PXE, GACI, and DCC result from a deficit in PPi production [18,19], CALJA is caused by increased PPi degradation [10,11] (Figure 1). The explanation of these phenotypes places ABCC6 as an upstream modulator of an extracellular purinergic pathway that, among other things, inhibits mineralization by regulating the Pi/PPi ratio in connective tissues (Figure 1). Although it has been recently claimed that ABCC6 acts downstream of NPP1 [20], this hypothesis is highly controversial in the field [21] and contradicts most studies [8,9,11,18]. This pathway (Figure 1) and the molecular cascade leading to PPi generation and TNAP inhibition offers many opportunities for therapeutic interventions in the case of PXE and also GACI.

At present, only palliative treatments to alleviate some symptoms exist for both PXE and GACI [22,23,24]. Extensive studies on the mechanism behind calcification has resulted in several novel approaches to treating PXE and GACI. The therapeutic solutions envisioned and tested in animals include strategies focusing on two distinct aspects of the ABCC6 pathway: (1) the correction/replacement/inhibition of dysfunctional genes/proteins involved in the calcification pathway [9,18], and (2) supplementation therapies with exogenous compounds. Drugs targeting ABCC6, NPP1 and TNAP aim at correcting parts of the ABCC6 pathway, whereas exogenous compounds such as magnesium, vitamin K, bisphosphonates (PPi analogs), PPi and recently phytic acid are intended to directly inhibit calcification.

In this article, we provide an overview of ABCC6 as a key regulator of ectopic calcification in PXE (and GACI). We will discuss possible treatment options that have been explored in recent years and speculate as to what future treatments might be. Because ABCC6 and NPP1 are functionally related proteins interchangeably causing PXE and GACI [17], we address herein some aspects of GACI and NPP1, which can be relevant for therapeutic interventions.

## 2. The Pathologies Associated with ABCC6 and ENPP1 Deficiencies

### 2.1. Pseudoxanthoma Elasticum

Pseudoxanthoma elasticum (PXE) is a rare disorder with progressive ocular, vascular and skin abnormalities that result from the accumulation of morphologically abnormal calcified elastic fibers [25]. The skin manifestations are the most prevailing characteristic of PXE, but the ocular and cardiovascular symptoms are responsible for the morbidity of the disease.

The first description of the clinical signs of PXE appeared in the literature more than a century ago [26,27,28]. Is it Darier who proposed the term “pseudoxanthome élastique”, which remains largely used to this day as pseudoxanthoma elasticum [29].

#### 2.1.1. Dermal Manifestations

Skin lesions are generally the first signs of PXE to be observed during childhood or adolescence and often progress slowly and unpredictably. Therefore, a dermatologist frequently makes the initial diagnosis. The accumulation of abnormal calcified elastic fibers in the mid-dermis produces yellow-hued papules and plaques and laxity with loss of elasticity. These lesions can be seen on the neck, axilla, antecubital fossa, popliteal fossa, groin, and periumbilical area [25,30,31,32].

#### 2.1.2. Ocular Manifestations

Ocular lesions in PXE are due to the accumulation of abnormal elastic fibers in the Bruch’s membrane, resulting in angioid streaks [33]. Angioid streaks are completely asymptomatic and can remain undetected until later in life when retinal haemorrhages occur. The majority of PXE patients will develop ocular changes during their second decade of life. Bilateral angioid streaks are normally seen as linear gray or dark red lines with irregular serrated edges lying beneath normal retinal blood vessels and they represent breaks in the Bruch’s membrane. The elastic laminae of the Bruch’s membrane is located between two layers of collagen and lies in direct contact with the basement membrane of the retinal pigmented epithelium (RPE) and the capillaries of the choroid. Angioid streak formation is likely the direct result of mineralization of elastic fibers in Bruch’s membrane. As a consequence of the loss of structural integrity to the Bruch’s membrane, PXE patients progressively develop choroidal neovascularization, which can lead to hemorrhagic detachment of the fovea and retinal scarring. Optic nerve drusen may also be associated with angioid streaks and results in visual field deficits.

It is worth noting that a minor yet important palliative treatment exists for some of the ocular manifestations of PXE [24] and more details are provided below (*Cf.* Section 6.2).

#### 2.1.3. Cardiovascular Manifestations

The common cardiovascular complications of PXE are due to the presence of abnormal calcified elastic fibers in the internal elastic lamina of medium-sized arteries. The broad spectrum of phenotypes includes peripheral arterial disease (PAD), resulting from restrictive vascular calcification in the lower limbs, increased susceptibility to atherosclerosis [34,35,36], intermittent claudication, and possibly myocardial infarction and hypertension. The fibrous thickening of the endocardium and atrioventricular valves can also result in restrictive cardiomyopathy and/or mitral valve prolapse and atrial septal aneurysm [37]. A cardiac evaluation of French PXE patients revealed sporadic cases of left ventricular hypertrophy, aortic stenosis, and valvulopathies. Overall in this cohort, PXE does not appear to be associated with frequent cardiac complications. However, the development of cardiac hypertrophy in old *Abcc6*^−/−^ mice suggests that aging PXE patients might be susceptible to late cardiopathy [38].

Extra cardiac downstream effects of arterial mineralization include renovascular hypertension, gastrointestinal bleeding and central nervous system complications (such as stroke and dementia) [30,39].

A comprehensive analysis of the arterial consequences (macro- and micro-arterial beds were investigated) of ABCC6 ablation in mice was published in 2014 [40]. This report revealed scattered arterial calcium depositions as a result of osteochondrogenic transdifferentiation of vascular cells as mice age (as seen via increase *Runx2* expression). Lower elasticity and increased myogenic tone without major changes in agonist-dependent contraction was found in older *Abcc6*^−/−^ mice suggesting reduced control of peripheral blood flow, which in turn may alter vascular homeostasis which is not unlike the PAD observed in PXE patients.

#### 2.1.4. Renal Manifestations

Renal involvement in PXE has received little attention in the literature until recently. Few cases of kidney stones in PXE patients have been described, whereas classic nephrocalcinosis has only been reported in sporadic cases [41,42,43]. More recent data suggested that nephrolithiasis was an unrecognized and prevalent feature of PXE [44]. The first in depth analysis of renal manifestations in a cohort of 113 French PXE patients showed a history of kidney stones in 40% of patients, which is much higher than the general population. Computed tomography scans revealed evidence of significant papillary calcifications (Randall’s plaques) [45,46].

Remarkably, despite the presence of mineralized elastic fibers in pulmonary tissues, there is no lung phenotype associated with PXE [47,48].

### 2.2. Generalized Arterial Calcification of Infancy (GACI)

Generalized arterial calcification of infancy (GACI [MIM 208000]) is a very rare autosomal recessive disorder characterized by calcification of arterial elastic fibers and associated fibrotic myointimal proliferation of muscular arteries and arterial stenosis [49]. Severe vascular calcification causes hypertension, myocardial ischemia, and congestive heart failure [17]. The majority of patients die within the first six months of life [50,51]. However, patients treated with bisphosphonates can experience a more favorable outcome [52,53]. In addition to vascular mineralization, neonatal patients also present periarticular soft-tissue calcifications. More mildly affected patients may also develop hypophosphatemic rickets [53,54,55].

### 2.3. PXE and GACI Are Different Clinical Manifestations of a Phenotypic Continuum

PXE is primarily caused by ABCC6 deficiency, while GACI patients typically present mutations in the *ENPP1* gene. However, some GACI patients only carry *ABCC6* mutations and typical PXE manifestations can be associated with *ENPP1* mutations. The clinical and molecular genetic characterization of PXE and GACI [16,17] has suggested that ABCC6 and NPP1 are functionally related [56] and give rise to overlapping phenotypes with GACI being a severe and acute form of PXE and vice versa [8,16,57].

Calcification of joints and arteries (CALJA, OMIM#211800) is outside of the scope of this review. However, it is worth mentioning briefly because its molecular etiology is intimately related to that of PXE and GACI. CALJA is due to mutated CD73 (encoded by *NT5E*) [10], which is functionally downstream to NPP1 (Figure 1). The disease is characterized by vascular calcification in the lower limbs and periarticular mineralization. CALJA is caused by enhanced PPi degradation [10,11] resulting from reduced adenosine signaling and abnormal activation of tissue non-specific alkaline phosphatase (TNAP/*ALPL*) [11,20] (Figure 1).

### 2.4. Thalassemia

For the most part, PXE is a monogenic disease caused by mutations in *ABCC6*. However, other than the cases associated with *ENPP1* mutations [17], PXE manifestations can arise from multifactorial inheritance [58,59], environmental exposure [60,61,62,63] or secondary to β-thalassemia and sickle cell anemia [64]. β-thalassemia (MIM 141900) is a monogenic disorder caused by mutations in the β-*globin* gene that leads to the underproduction of β-globin chains. The stoichiometric excess of α-chains unbound to β-globin is unstable and precipitates in red blood cell precursors causing the ineffective erythropoiesis. In the past decade, it has become apparent that a large number of Mediterranean patients affected by β-thalassemia or sickle cell anemia also develop manifestations similar to PXE [64]. β-thalassemia and PXE are distinct genetic disorders, yet, the ectopic mineralization phenotype seen in β-thalassemia and sickle cell patients is clinically and structurally identical to inherited PXE [65,66,67,68] and arises independently of *ABCC6* mutations [69]. Based on studies with a mouse model of β-thalassemia (*Hbb^th3/+^*), it was suggested that the β-thalassemia patients could have a suboptimal endowment of ABCC6 expression in liver (and possibly reduced PPi production), thereby increasing susceptibility to ectopic mineralization in a PXE-like manner. If this is indeed the case, then β-thalassemia and sickle cell patients could benefit from several treatment options developed for the inherited forms of PXE.

## 3. The Structure and Molecular Function of ABCC6

The ATP-binding cassette (ABC) family represents the largest group of transmembrane proteins. These proteins bind ATP and use its energy to drive the efflux and transport of various molecules across cell membranes. ABC transporters are classified based on the sequence and organization of their ATP-binding domains. The human ATP-binding cassette (ABC) gene family consists of 48 members divided into seven subgroups, A through G. Genetic changes in at least 14 of these genes cause heritable diseases [70,71]. About a third of all of these ABC transporter-related diseases are linked to genes from the single sub-group C, which includes ABCC6 and PXE but also the well-known cystic fibrosis associated with *ABCC7* mutations.

### 3.1. ABCC6 Structure

ABCC6 consists of 1503 amino acids with an approximate molecular weight of 165 kD without glycosylation. This transporter protein possesses three transmembrane segments with 5, 6, and 6 membrane-spanning regions, respectively, and two typical ABC domains involved in the binding and hydrolyzing of ATP used for conformational changes and transport activity. A 3D model of ABCC6 was successfully generated using X-ray structure of the *S. aureus* Sav1866 export pump [72]. Fülöp et al. used this model of ABCC6 and the distribution of PXE-causing mutations to demonstrate the strict relevance of the transmission interface (ICL-ABC contacts) as well as the ABC-ABC domain contacts for the function of the transporter [73].

### 3.2. ABCC6 Is an Efflux Pump

Transport studies in vesicles isolated from Sf9 cells expressing the rat *Abcc6* identified the anionic cyclopentapeptide and endothelin receptor antagonist BQ-123 as substrates [74]. ATP binding and hydrolysis by rat ABCC6 has been demonstrated in a yeast expression system [75]. Using a similar approach with the human ABCC6, Ilias et al. demonstrated ATP binding and ATP-dependent active transport of the glutathione conjugates leukotriene C_4_ and *N*-ethylmaleimide *S*-glutathione and of the cyclopentapeptide BQ-123 [76]. Probenecid, benzbromarone and indomethacin specifically inhibited transport of *N*-ethylmaleimide *S*-glutathione [77]. Another study confirmed ATP-dependent transport of BQ123 and leukotriene C_4_ by human ABCC6 and also identified *S*-(2, 4-dinitrophenyl) glutathione as an in vitro substrate. Analysis of the drug sensitivity of ABCC6-transfected cells revealed low levels of resistance to etoposide, teniposide, doxorubicin, and daunorubicin, indicating that ABCC6 is able to confer low levels of resistance to certain anticancer agents [78]. However, this function appears unlikely to play a role in the pathophysiology of PXE and thus has limited bearing on the possible therapeutic solutions. Note that the actual endogenous substrate(s) and the precise molecular mechanism by which ABCC6 achieves the efflux of ATP and other nucleotides/nucleosides are still unknown.

### 3.3. Cellular Localization of ABCC6

ABCC6 is firmly associated with the basolateral membrane of hepatocytes in mice, rats, and humans [74,79,80], as well as in the proximal kidney tubules [81,82]. Although a report challenged the well-established cellular localization of ABCC6 [83], it is interesting to note that disease-causing missense *ABCC6* mutations can lead to aberrant cellular localization [80].

### 3.4. NPP1 Structure

NPP1 is a membrane bound enzyme consisting of 840 amino acids (just under 100 kDa) that belongs to the ecto-nucleotide pyrophosphatase/phosphodiesterase (ENPP) family. NPP1 is a type II transmembrane glycoprotein comprised of two identical disulfide-bonded subunits. The polypeptide has a short N-terminal cytoplasmic domain followed by a transmembrane region. The extracellular structure consists of two somatomedin B (SMB)-like domains (SMB1 and SMB2), a phosphodiesterase domain and a nuclease-like domain [84]. The SMB-like domains are disordered and do not interact with the catalytic domain. The mapping of disease-causing mutations highlights the functional significance of the interaction between the catalytic and nuclease-like domains. It is the SMB2 domain of NPP1 that interacts with the insulin receptor and carries out physiological functions other than the regulation of mineralization [85].

## 4. Mutations in ABCC6 and ENPP1

### 4.1. ABCC6

Over 400 mutations (https://www.ncbi.nlm.nih.gov/clinvar, accessed on 3 February 2021) have now been identified in *ABCC6*. Most of these are single nucleotide changes leading to a panel of missense, nonsense, splice site, and frameshift mutations, in addition to small and large insertions/deletions. Missense variants are found in areas critical to the stability and/or the function of the protein. Based on a small but representative number of ABCC6 mutants, two possible molecular consequences to *ABCC6* mutations were described: (1) transport deficiency linked to the failure to hydrolyze ATP and (2) abnormal protein folding leading to intracellular retention and/or reduced trafficking. The latter has been the focus of studies designed to rescue trafficking by re-purposing the drug 4-phenylbutyrate (*Cf.* Section 6.3.2). Transport deficiency and abnormal trafficking are likely the reasons behind the loss of physiological function and provide a reasonable explanation for the lack of phenotype–genotype correlation in PXE [86,87,88].

About 40% of all *ABCC6* gene mutations in patients with PXE consist of premature termination codon mutations and account for 25% of all mutations in PXE patients [88]. However, two mutations are recurrent in the Caucasian population, p.R1141X and g.del23-29, which account for up to ~45% of all mutant alleles [87,88].

These ABCC6 mutations have consequences for the entire calcification pathway, as evidenced by the downregulation of ENPP1 and NT5E gene expression in the absence of ABCC6 [11,18], the reduced plasma levels of PPi [8], and the activation of TNAP as a consequence of lowered adenosine production [11,20].

### 4.2. ENPP1

On the ClinVar database, there are nearly 100 ENPP1 pathological variants reported. These variants present a similar profile to that found in ABCC6, with a large majority being single nucleotide substitutions leading to missense, nonsense, splice site, and frameshift mutations, plus some deletions and duplications. The main consequence of these disease-causing variants, which affects all critical parts of the protein, is the inactivation of the enzyme resulting in the failure to convert ATP (or ADP) into AMP and PPi. As NPP1 is the only enzyme that generates PPi, plasma levels drop to near zero in its absence [89].

## 5. Animal Models

### 5.1. The PXE Mice

Two lines of *Abcc6* knockout (*Abcc6*^−/−^) mice were generated independently [90,91]. In these *Abcc6*^−/−^ mice, *Abcc6* exons 15 to 18 were deleted. Both mouse lines lack the ABCC6 protein and develop identical calcification phenotypes that are consistent with the human PXE condition. The mice breed normally and have a life span of about 25+ months. These animals display spontaneous mineralization in vascular, ocular, and renal tissues, as well as in testes and vibrissae in the whiskers. The earliest evidence of mineralization occurs at 5–6 weeks of age in the capsules of vibrissae. The calcification in vibrissae is progressive and quantifiable and thus serves as a reliable marker of disease progression [92]. Heterozygous *Abcc6*^+/−^ mice do not develop any calcification. Similar to their human counterpart, *Abcc6*^−/−^ mice have lowered plasma PPi [9,18] altered lipoproteins [93,94], develop Randall’s plaques, and have low urinary PPi excretion [45,46]. These animals have been invaluable for understanding the pathobiology of PXE and to test crucial pathophysiological hypotheses [95,96] that in vitro approaches could partially address [97]. These mice were the ultimate tool that allowed the development of therapeutic solutions for PXE patients [18,19,92,98,99].

### 5.2. The Murine DCC Phenotype and Other PXE Mouse Models

In the past years, two groups of investigators have found that ABCC6 deficiency causes an acute and inducible dystrophic cardiac calcification phenotype (DCC) affecting several strains of inbred mice, including C3H/HeJ, 129S1/SvJ, and DBA/2J [12,14,100]. DCC is an autosomal recessive trait that was described decades ago [101,102]. DCC can either occur spontaneously over the long-term, be initiated by a specific dietary regimen, or be triggered into an acute phenotype by direct injury [103,104] or ischemia [13]. Of note, arteries (most notably the aorta) as well as skeletal muscles, are also susceptible to dystrophic calcification [100,105] (Le Saux et al., unpublished results). DCC is caused by a single *Abcc6* gene mutation in C3H/HeJ, 129S1/SvJ, and DBA/2J mice [12], while it is absent in C57BL/6J mice that are DCC-resistant. Note that the *Abcc6*^−/−^ mice that have been used thus far were all backcrossed into C57BL/6J. This is important as there are 3 other minor loci affecting the penetrance and the expression of DCC mapping to chromosomes 4, 12, and 14 [104].

**Figure 2:**
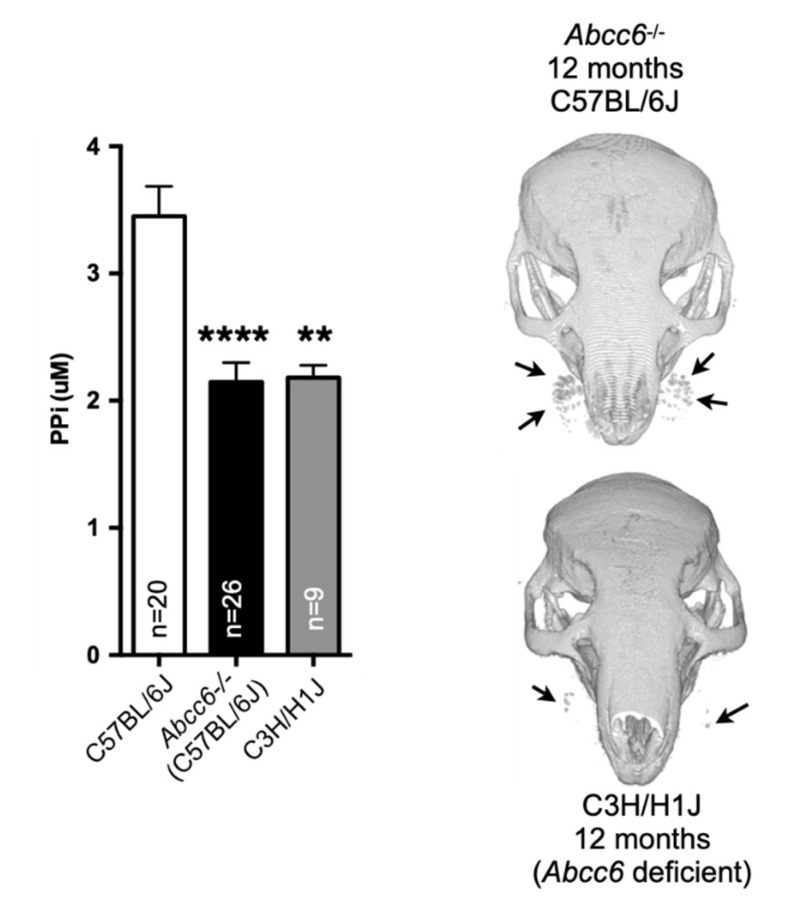
Plasma pyrophosphate levels do not correlate with the calcification phenotype in mice. Plasma pyrophosphate levels in *Abcc6*^−/−^ mice (C57BL/6J background), and in C3H/H1J mice with a naturally occurring *Abcc6* mutation are virtually identical and significantly lower than wild type C57BL/6J mice. However, at 12 months of age, *Abcc6*^−/−^ mice present a much more pronounced vibrissae calcification as shown on this μCT scan rendering (right, arrows). Plasma PPi results are shown as means +SEM. *p*-values were determined by Student’s *t*-test. ** *p* < 0.01, **** *p* < 0.0001. Derived from [111].

Remarkably, DCC-susceptible C3H/HeJ mice develop an attenuated version of the murine PXE phenotype as compared to the *Abcc6*^−/−^ animals, while the DBA/2J mice present little or no manifestations [106]. It is interesting to note that the murine PXE manifestations reported in KK/H1J mice are remarkably severe [107]. These mice show systemic age-dependent ectopic mineralization, hyperplasia, and fibro-osseous lesions [108]. Calcification mostly affects vibrissae (as for *Abcc6*^−^^/^^−^ animals), but also the heart, the lung and many other organs to a lesser degree. Pancreatic islet hyperplasia was observed as well as fibro-osseous lesions in several bones. More interesting is that these strains of mice carry the exact same *Abcc6* gene mutation and have similar plasma PPi levels (Figure 2), which clearly underlie the possible role of other factors such as the environment [19] and/or modifier genes in the development of ectopic calcification [109,110].

### 5.3. The PXE Rat

PXE mouse models have been excellent research tools but their small physical size can be an impediment to using certain investigative tools and techniques such as perfusion or organ transplantation. In a recent report, Li et al. described the generation of *Abcc6*^−/−^ rats using zinc finger nuclease (ZFN) technology [112]. These animals displayed a calcification phenotype similar to that of *Abcc6*^−/−^ mice, with mineralization in the skin (vibrissae), eyes (Bruch’s membrane), kidneys, and arterial beds. Plasma PPi levels were depleted by more than 60%, which is consistent with *Abcc6*^−/−^ mice and PXE patients. Using in situ perfusion experiments, the authors observed a near abolition of PPi levels in liver (and kidney) perfusates, which has also been described in mice [9,18].

### 5.4. The “PXE” Zebrafish

Rodent models present remarkable similarity to human diseases and have proven extraordinarily useful. However, like all models, these systems have limitations, i.e., a relatively long life span and the associated cost of development and maintenance. Zebrafish are models that address some of these limitations and provide higher “n” numbers for observation and quantification. Zebrafish carry two orthologs of human *ABCC6* referred to as *abcc6a* and *abcc6b*. These genes encode proteins of 1507 and 1502 amino acids, respectively, which are similar to the human polypeptide at 1503 amino acids. *Abcc6a* and *-b* have ~50% identity and ~70% similarity with the human protein, respectively. The first attempt to generate a zebrafish model used morpholino technology, which is now progressively replaced by genome-targeting methods such as CRISPR/Cas9 because it has often been difficult to discriminate between specific and non-specific effects. Li et al., 2010 reported the expression profiles of both *abcc6a* and *abcc6b* and the lack of phenotype associated with *abcc6b* knockdown [113]. Injection of *abcc6a*-specific morpholinos induced cardiac and developmental malformations followed by death of the animals. This phenotype could be rescued with injection of mouse *Abcc6* cDNA, suggesting some evolutionary conservation in the physiological function of ABCC6. More recently, van Gils and co-workers described a CRISPR/Cas9 *abcc6a* knockout zebrafish [114]. This zebrafish model showed hypermineralization of the spine starting in the embryonic stage and progressing into adulthood with scoliosis, vertebral and rib mineralization and loss of normal bone architecture. These manifestations are not consistent with the human PXE condition and there was no obvious mineralization in the skin or in the eyes. Thus, the *abcc6a* zebrafish model develops an abnormal calcification phenotype but one that does not recapitulate the human PXE. A new zebrafish model was recently created using the transcription activator-like effector nuclease (TALEN) technique [115]. These animals displayed similar skeletal changes as well as calcification in the ocular Bruch’s membrane and a range of other previously unreported manifestations, such as cardiac fibrosis. Remarkably, this phenotype could be attenuated by vitamin K treatment, similar to what had been previously reported in 2015 with another zebrafish model [116].

Overall, zebrafish have shown some utility, notably in the characterization of the 4-phenylbutyrate treatment option for PXE [117], but their evolutionary distance and phenotypic divergence limit their usefulness for understanding the pathophysiology of PXE. However, they could be excellent preliminary testing tools for anti-calcification treatments.

### 5.5. The GACI Models

#### 5.5.1. GACI Mice

The first evidence of a link between defective *Enpp1* expression and abnormal mineralization was first demonstrated in ‘tiptoe walking’ (*ttw*/*ttw*) mice [118]. The various *Enpp1* deficient mouse models that exist present similar characteristics. The animals with a naturally occurring mutation (*ttw*/*ttw*) carry a homozygous nonsense variant in the *Enpp1* coding sequence [118], whereas another mouse model harbors a missense mutation [119]. The phenotype of these mice includes progressive ankylosing intervertebral and peripheral joint hyperostosis, as well as spontaneous arterial and articular cartilage calcification and increased vertebral cortical bone formation. Low plasma PPi and PXE-like calcification are fundamental characteristics of these mice [119,120]. These animals also have abnormal ossification, cardiovascular pathologies and altered glucose homeostasis and diabetes [121,122].

#### 5.5.2. The GACI Zebrafish

Similar to PXE and ABCC6, a zebrafish model of GACI was generated targeting the *Enpp1* gene [123]. Unlike the *abcc6a* KO counterparts, these mutant zebrafish developed ectopic calcifications similar to that seen in GACI and PXE models in a variety of soft tissues that included the skin, cartilage, heart, intracranial space, and the notochord sheet. Treatment with the bisphosphonate etidronate (a PPi analog) rescues some aspects of the phenotype. The report also noted that the expression of *Enpp1* in blood vessels or the floor plate of mutant embryos was able to rescue the notochord mineralization phenotype.

## 6. Rescue and Therapeutic Solutions

**Figure 3:**
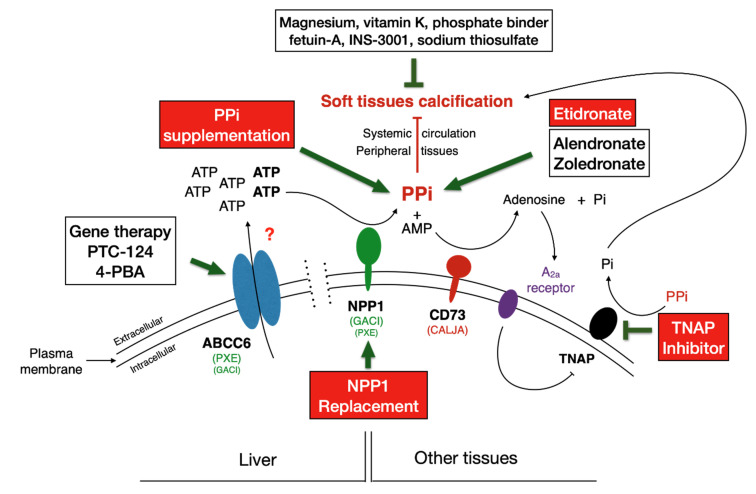
Tested therapeutic Interventions targeting different steps in the ABCC6 pathway to prevent calcification in PXE/GACI. Red boxes point to approaches that were tested in preclinical models and that are currently under human evaluation/trials.

In recent years, basic science has elucidated the molecular etiology underlying PXE [6,8,13,76,87,96,124,125], which has led to rapid advances in the development of many potential therapeutic options for PXE and GACI and the recent completion of three pilot clinical trials [18,19,20,80,99,117,126,127,128,129,130]. Among the several approaches (summarized in Table 1) that have been considered are enzyme replacement therapy, rescue drugs, and enzyme inhibitors, as well as exogenous compounds such as PPi. All target various steps in the ABCC6 pathway (Figure 3) with the goal of either slowing or reversing the progression of the disease. Figure 3 outlines many of these approaches and indicates the targeted parts of the molecular pathway. We discern two main categories of therapeutic solutions that have undergone development and testing, thus far. Most of these therapeutic solutions have been tested in pre-clinical animal models (mice and zebrafish) and a few phase I/II clinical trials. Beyond what is described in these paragraphs, note that there are larger scale trials in preparation and further drug development that are taking place based on past lessons and data. We cannot describe these ongoing efforts here for reasons of confidentiality or simply for lack of detailed information.

### 6.1. Treatment Outcome Conundrum

Before we begin describing treatment options, there is one important point to address. With the multiplicity of therapeutic solutions developed and tested, there has been strong consideration given by both scientists and physicians as to what clinical criteria should be used to reliably evaluate and quantify treatment outcomes for PXE patients. This debate, which has been ongoing for several years, has yet to be settled due to the complexity of the PXE phenotype as multiple organ systems (skin, eyes, cardiovascular, renal, etc.…) are affected with various degree of distribution, severity and considerable inter- and intra-familial variability [150,151,152]. Indeed, identical mutations can cause the relatively mild PXE or severe GACI [17] in humans but also in mice [111]. The lack of genotype/phenotype correlation only compounds the problem. However, a very recent clinical study performed with 289 PXE patients found that mixed genotype led to less severe arterial calcification and ocular manifestations than patients with two truncating *ABCC6* variants and suggested that environmental or modifier genes could also contribute to the still unexplained phenotypic variability in PXE [153]. The presence of modifier genes modulating calcification in PXE has been investigated in human and mice but their predictive values for clinical applications are not clear [109,110,147,150,154,155,156]. Moreover, PXE is a slow and chronic disease with stepwise progression of the phenotype occurring over a period of time measured in years, if not decades [157]. The progression of calcification in *Abcc6*^−/−^ mice mirrors that of humans and typically takes many months to develop [18,92]. PPi has been considered as a potential biomarker as it is central to the etiology of PXE [8] but its plasma levels only bring partial information as to the status of the disease [158] (See Figure 2). Furthermore, the daily and long-term natural variations of plasma PPi are not well known. Measuring calcification is an obvious approach but its quantification in a non-invasive or minimally invasive manner is not a trivial task. Invasive biopsies were tested in early trials in semi-quantitative evaluations [129,130]. However, these trials did not yield positive conclusions and it is possible that the quantification methodology combined with the heterogeneity of the manifestations may have contributed to the inconclusive results. The capacity of ^18^F-sodium fluoride (^18^F-NaF) positron emission tomography/computed tomography (PET/CT) to identify and evaluate metabolically active osteogenesis in skin and arteries in PXE patients has been demonstrated [159,160]. This method was applied in the recent TEMP clinical trial and was able to discern some phenotypical changes within a year following etidronate administration (*Cf.* Section 6.7.4) [126]. Although the potential of some novel methodology is being investigated, at this time [161], PET/CT scan is probably the best option available to evaluate treatment outcome in PXE patients, but it requires access to well-equipped medical centers. However, it is very likely that a combination of methodology and multiyear trials will be needed to achieve clear and objective results in the future.

### 6.2. Palliative Treatment: An Ocular Therapy for PXE

As described above, PXE leads to severe visual impairment as a consequence of choroidal neovascularizations resulting from angioid streaks. Visual loss is one of the PXE manifestations that impacts quality of life the most. Photodynamic therapy has been explored but was not found to be effective [162]. Because the monoclonal antibody against the vascular endothelial growth factor (VEGF–Bevacizumab) has been shown to be beneficial for the treatment of choroidal neovascularizations secondary to age-related macular degeneration [163], which present many similarities with PXE, Finger et al. performed a retrospective analysis with a small cohort of PXE patients to determine the effectiveness of repeated intravitreal injections of Bevacizumab on visual acuity and morphologic outcomes [24]. The results were unequivocal in showing that this therapy preserved ocular function in cases of advanced disease and improved function in early stages. As a result, anti-VEGF therapy has become a common symptomatic treatment for PXE patients.

### 6.3. Targeting ABCC6

ABCC6 deficiency causes a broad spectrum of manifestations beyond calcification that includes vascular malformation (rete mirabile, carotid hypoplasia) [164], dyslipidemia, and atherosclerosis [93,94], as well as ischemic stroke [165], inflammation [159], premature cellular senescence in hepatic cells and dermal fibroblasts [166,167], increased infarct size, and apoptosis [168]. ATP released by ABCC6 and the nucleotides/nucleosides generated downstream by the ectonucleotidases NPP1 and CD73 regulates cellular signaling towards P2 (nucleotides) and P1 (Ado) receptors, which have a wide range of physiological influences that could well explain these other manifestations [124]. The primary advantage and benefit from therapies that directly aim at restoring ABCC6 function would be the potential rescue of the full spectrum of manifestations, not just calcification.

#### 6.3.1. PTC-124

About 400 distinct disease-causing variants have so far been reported in the *ABCC6* gene (https://www.ncbi.nlm.nih.gov/clinvar, accessed on 3 February 2021). A little more than 1/3 of all variants are nonsense that most likely result in nonsense-mediated decay or possibly truncated ABCC6 proteins. The most common and recurrent variant (and the first to be identified) is the nonsense p.R1141X, which accounts for ∼30% of all pathogenic alleles in PXE patients of Caucasian descent [169,170].

Correct protein synthesis is at the core of biological processes of all living organisms. Therefore, many compounds with the ability to overcome premature termination codons (PTC) have been studied over the years with the hope of restoring full-length protein synthesis as a therapeutic approach for heritable diseases [171]. Indeed, about 12% of human genetic disorders are caused by nonsense mutations, leading to the generation of PTC. This strategy was tried previously with cystic fibrosis patients. In a phase II clinical trial, these patients were given 1,2,4-oxadiazole (PTC-124), a non-aminoglycoside nonsense mutation suppressor, with some success [172]. However, follow-up phase III trials were relatively unsuccessful [173]. This drug is approved for clinical use by the European Medicines Agency (EMA) to treat Duchenne muscular dystrophy but not by the Federal Drug Administration in the US. It is commercialized under the names of Ataluren or Translarna and can be delivered orally. It has not yet been tried clinically for PXE patients.

One preclinical study examined the efficacy of PTC-124 in a “PXE” zebrafish morpholino model. Zhou et al. utilized HEK293 cells transfected with ABCC6 expression vectors harboring seven different PXE-nonsense mutations, including the most common stop codon mutation, p.R1141X, to evaluate whether PTC-124 could facilitate read-through of these variants [131]. Using immunostaining, they found that the amount of full-length protein synthesis was increased after 72 h of treatment with PTC-124 at a concentration of 5 µg/mL. To test the functionality of these potential full-length read-through ABCC6 proteins, they employed a zebrafish morpholino rescue system. As PTC-124 primarily replaces UAG PTC with tRNA corresponding to Trp, Cys, and Arg (here replaced by Gly), the authors of this study showed that these three possible read-through *ABCC6* variants rescued an *Abcc6a* morpholino-induced phenotype in zebrafish. Of note, in the *ABCC6* mutation database, a single missense variant affecting codon R1141 is reported as p.R1141Q and is classified as benign. It is therefore possible that substitutions at the R1141 site may not have negative impact. Despite these encouraging results, the study did not address calcification and there has been no follow-up study in mouse models of PXE.

One important argument for using PTC-124 would be restoration of the full physiological function of the entire ABCC6 → NPP1 → NT5E⊣TNAP pathway (Figure 1 and Figure 3) as the *Enpp1*, *Nt5e* and *Alpl* genes are affected in the absence of *Abcc6* [11,18]. In addition, a large fraction of *ABCC6* disease-causing variants are nonsense, notably the R1141X variant, which is particularly prevalent in Caucasian PXE and GACI patients [169,170]. Another advantage favoring PTC-124 is its oral delivery and that only one allele needs to be targeted, as PXE and GACI are recessive diseases. However, these advantages are also limitations, as treatment would only be restricted to those carrying nonsense alleles.

#### 6.3.2. 4-Phenylbutyrate (4-PBA)

Drug repurposing has gained attention over the past years to cut development cost in part because the safety of the drugs is known and the risk of failure is reduced. This is particularly advantageous for rare diseases like PXE and GACI. 4-phenylbutyrate (4-PBA) is an aromatic fatty acid normally used to treat urea cycle disorders and thalassemia [174,175,176]. One of its metabolites, phenylacetylglutamine, contains the same nitrogen amount as urea, and is thus an alternative to capturing excess nitrogen before excretion by the kidneys. 4-PBA is classified as an orphan drug commercialized under the names of sodium phenylbutyrate in the U.S. and BUPHENYL or AMMONAPS in other countries. Glycerol phenylbutyrate (RAVICTI) is a related compound. It is a triglyceride pro-drug containing three molecules of 4-PBA linked to a glycerol backbone. RAVICTI is an FDA-approved alternative to sodium 4-PBA, recommended for pediatric use due to its improved palatability [177]. 4-PBA also influences the transcription of endoplasmic reticulum chaperones [178] and this off-target property has been exploited to treat patients with diseases caused by improper translocation of proteins to the plasma membrane, including other ABC transmembrane proteins [179,180,181,182].

The repurposing of 4-PBA for use in PXE has been explored in vitro as well as with *Abcc6*^−/−^ mice [80,99,117]. 4-PBA was first tested to determine if it could restore the normal cellular trafficking in vitro and in vivo of 10 frequently occurring disease-causing *ABCC6* missense mutants as well as phenotypic rescue in zebrafish. Seven of the mutants were transport-competent but were retained intracellularly. 4-PBA successfully restored the plasma membrane localization and functionality of four of these ABCC6 mutants thus providing the first evidence that 4-PBA therapy was possible for selected patients with PXE and GACI.

In a follow-up study, the combination of 4-PBA treatment and the transient expression of human *ABCC6* mutants in the liver of *Abcc6*^−/−^ mice was tested against the DCC phenotype, which is a reliable indicator of ABCC6 function [99]. Treatments restored the physiological function of human *ABCC6* mutants and inhibited calcification, but interestingly, failed to restore PPi levels in plasma. However, collectively these studies demonstrated the credibility of 4-PBA for the treatment of both PXE and GACI [99].

The main advantage of 4-PBA is similar to that of PTC-124 and this drug has been in clinical use for several decades. This would certainly facilitate and expedite clinical trials if it were to be tested in eligible PXE/GACI patients. The limitations of 4-PBA would also be similar to those of PTC-124, being an allele-specific drug that can only be applied to *ABCC6* missense mutants with verified sensitivity to the drug. However, the premise of potentially superior approaches focused on restoring PPi levels have supplanted both PTC-124 and 4-PBA and no clinical studies are currently envisioned for either drug.

### 6.4. Targeting NPP1

As most cases of GACI are caused by mutations in *ENPP1*, enzyme replacement therapy has been proposed as a potential strategy to counteract the clinical manifestations of the disease. NPP1 is a key enzyme in the calcification pathway, converting extracellular ATP into adenosine monophosphate and PPi. There has been success with this strategy in preclinical studies. Albright et al. demonstrated that replacement therapy with soluble recombinant human NPP1 (rhNPP1-Fc) successfully reduced ectopic mineralization, improved plasma PPi levels, and prevented mortality in a rodent GACI model [132]. Another study evaluating cardiac outcomes found recombinant NPP1 reduced not only aortic calcification, but also improved cardiovascular function and blood pressure [133].

GACI presents manifestations beyond calcification that can potentially be attributed to the influence of NPP1 enzyme activity on extracellular purinergic metabolism [89], which is consistent with what has been found for ABCC6 and PXE [124]. In a recent study, Nitschke and co-workers showed that nearly ¾ of GACI patients display arterial stenoses due to intimal proliferation of vascular smooth muscle cells (VSMC) [89]. This phenotype can be reversed effectively in culture and in vivo with rhNPP1-Fc or with adenosine. However, neither PPi nor the bisphosphonate etidronate (an off-label treatment for GACI) had an effect on VSMC proliferation. This is an important finding as it pertains directly to PXE. Indeed, NPP1, which is immediately downstream of ABCC6, is not only essential for the generation of extracellular PPi but also for the cleavage of extracellular ATP into adenosine via the action of CD73 (Figure 1 and Figure 3). Remarkably, CALJA patients with *NT5E* mutations (and inactive CD73) develop similar vascular obstructions in the lower extremities in adulthood [10].

rhNPP1-Fc has yet to be tested in rodent models of PXE; however, there are some indications that it could potentially be used in this case as well. It has been observed, even with complete ABCC6 deficiency, that there are fairly high residual plasma levels of PPi (~40%), indicating that other sources of extracellular ATP exist [9,112]. However, it is possible that this strategy would require combination therapy to increase ATP release from cells other than hepatocytes and replace NPP1 in order to restore plasma PPi levels. However, preclinical studies are needed to demonstrate the viability of this therapeutic strategy for PXE.

### 6.5. Targeting TNAP

Alkaline phosphatases are a group of isoenzymes located at the cell surface catalyzing the hydrolysis of organic phosphate esters in a variety of phosphate-containing physiological compounds. They contribute to a range of physiological functions such as DNA synthesis, in addition to regulating calcification [183,184,185,186]. Alkaline phosphatases are classified as tissue-specific and tissue non-specific. The isoenzymes found in the intestine, placenta, and germinal tissue are tissue-specific, whereas the tissue non-specific (TNAP), often referred to as the liver/bone/kidney alkaline phosphatase, is found in these and other tissues. These alkaline phosphatases are encoded by four different genes and each has distinct functions.

The first evidence of a physiological connection between TNAP and a pathology closely related to PXE was reported in 2011 by St Hilaire et al. [187], even though a molecular connection was established a few years later [10,168]. This was followed by a more elaborate series of experiments using cell culture and several mouse models targeting the transporter/enzymes of the ABCC6 pathway (Figure 1 and Figure 3) [20]. Using primary skin fibroblasts from PXE patients with confirmed *ABCC6* mutations, Ziegler et al. found that these cells had elevated *ALPL* gene expression and associated TNAP enzymatic activity as compared to controls. The authors also investigated the effects of a TNAP inhibitor, SBI-425 (30 mg/kg/day), administered in the food of *Abcc6*^−^^/^^−^ mice vs. etidronate (240 mg/kg/day) or normal chow starting at 6 weeks of age for 14 weeks. MicroCT scans at 20 weeks revealed significant attenuation of calcification in both the mice treated with the TNAP inhibitor or etidronate. Furthermore, SBI-425 inhibited TNAP activity, whereas etidronate did not. More promising still as a potential therapeutic, *Abcc6*^−/−^ mice aged to 20 weeks and treated with the TNAP inhibitor did not show the same progressive calcification as control animals, although the treatment did not reverse existing calcification in this experimental context. Remarkably, Ziegler and colleagues found that plasma PPi levels did not increase after SBI-425 treatment, despite decreased plasma TNAP activity. This is somewhat surprising as PPi concentration in the plasma of *Nt5e*^−/−^ mice is significantly reduced [188]. Indeed, the lack of CD73 (NT5E) function leads to less adenosine and higher TNAP activity, resulting in higher rates of PPi hydrolysis. This apparent discrepancy is consistent with other circumstantial evidence [111,144], suggesting that plasma levels do not adequately reflect the steady state levels of PPi in connective tissues where it is most relevant.

A follow-up study used a slightly different approach to explore the role of TNAP in the calcification phenotype of PXE and GACI using *Abcc6*^−/−^*Alpl*^+/−^ double-mutant mice and *Enpp1*^−/−^ animals [127]. Mice heterozygous for *Alpl* in an *Abcc6*-deficient background showed both reduced plasma TNAP activity and mineralization as compared to controls, and the administration of SBI-425 led to similar results. By contrast, SBI-425 treatment of *Enpp1*^−/−^ mice did not produce any significant change in mineralization.

Overall, these studies demonstrated that inhibition of TNAP is a convincing treatment strategy for PXE (but maybe not for GACI). In fact, it is convincing enough that a pharmaceutical company is investing in this approach.

### 6.6. Gene Therapy

In 2010, an esoteric approach was explored based on the observation that plasma levels of fetuin-A in patients with PXE as well as in *Abcc6*^−/−^ mice are reduced by up to 30%. Fetuin-A is a circulating “hepatokine” predominantly synthesized in the liver, which possesses diverse physiological functions, including bone metabolism regulation, vascular calcification, and insulin resistance. Because fetuin-A is able to form complexes with calcium and phosphate ions, it acts as an inhibitor of ectopic calcification. Mice deficient in this glycoprotein show systemic calcification of soft tissues [134]. In this study [135], a fetuin-A cDNA was transiently expressed in the liver of *Abcc6*^−/−^ mice and resulted in an approximately 70% reduction of whisker calcification. However, the positive effects on calcification were not persistent.

More recently, the transient expression of the human *ABCC6* in mouse liver was shown to have a positive effect on DCC [13], while permanent expression via transgenes produced remarkable effects on ectopic calcification in several tissues (whiskers, kidneys, heart), despite having only modest effects on plasma PPi [18].

A more elaborate methodology was explored with the intravenous administration to *Abcc6*^−/−^ mice of a recombinant adenovirus carrying a cDNA encoding the normal human ABCC6 [136]. The mice showed high-level expression of the human ABCC6 in liver for several weeks post-delivery. This resulted in the normalization of plasma pyrophosphate levels. For sustained expression up to three repeated adenovirus injections 4 weeks apart were also tested with success. At the time of sacrifice the mice were 4–5 months old and showed very limited signs of mineralization in vibrissae. By contrast, treatments applied to older mice (11 months old) had no effect on existing mineralization. The human species C adenovirus serotype 5 (Ad5) used in this study is one the most frequently used gene delivery systems in animal and clinical studies [189] and presents a high predilection for liver transduction, which is the primary site of expression of ABCC6 [79,81]. This proof-of-concept study suggested that adenovirus-mediated *ABCC6* gene delivery may be possible to treat PXE and ABCC6-related GACI patients, when gene therapy, especially one targeting the liver, has reached sufficient maturity for clinical use.

### 6.7. Supplementation Therapies for Direct Inhibition of Calcification

Treatments with exogenous compounds such as magnesium, bisphosphonates (PPi analogs), and PPi are focused on reducing or counteracting the excessive susceptibility to mineralization found in PXE and GACI patients and to slow or interrupt the clinical progression of the disease.

#### 6.7.1. Calcium and Phosphate

Quickly after the generation of mouse models for PXE [90,91], several studies were initiated to look at the effect of dietary minerals (calcium, phosphate, magnesium) that could influence ectopic mineralization. Interestingly, a first attempt at altering the level of dietary calcium fed to *Abcc6*^−/−^ mice produced no significant effect on the calcification phenotype [138], mostly likely because plasma calcium levels are normally kept within a narrow range. However, changes to dietary minerals, notably phosphate, produced significant effects on soft tissue calcification in *Abcc6*^−/−^ mice [137]. These results prompted the exploration of phosphate binders as therapy. However, experimental testing in mice and human PXE patients showed this approach to be ineffective [130,137].

#### 6.7.2. Magnesium

Magnesium (Mg^2+^) is another mineral that inhibits the formation of apatite. In healthy individuals, serum magnesium concentrations are carefully balanced in a narrow range [190]. The kidneys are the main organs controlling systemic Mg^2+^ homeostasis, where its transport is highly regulated by hormonal and other intrarenal factors [191]. Bones are the main reservoir of Mg^2+^, holding ~60% of the total body content. Mg^2+^ in bones is primarily embedded at the surface of the apatite crystals [192]. Because of its inhibitory properties towards calcification and oral bioavailability, Mg^2+^ supplementation appeared to be a good option to decrease soft tissue calcification in pathologies with calcification such as chronic kidney disease (CKD) [193], but also in PXE, as both share some phenotypical characteristics [194]. Preliminary testing in *Abcc6*^−/−^ mice with increased dietary Mg^2+^ showed results [138,139] encouraging enough that a prospective human clinical trial was initiated (NCT01525875). However, the translation to humans proved to be inconclusive [129], probably because the dosages used in animal models exceeded what could actually be tolerated in humans and/or as a result of the method for phenotype evaluation methodology (*Cf.* Section 6.1). Therefore, Mg^2+^ supplementation is probably not a viable treatment strategy, at least on its own.

#### 6.7.3. Vitamin K

Normal bone mineralization is a highly regulated process, requiring a fine balance between inhibitors and promoters of calcification [195,196]. Among these regulators of mineralization, Matrix Gla Protein (MGP) and osteocalcin (OC) are two proteins reliant on carboxylation for activation and function [196,197]. Gheduzzi et al. described in 2007 high levels of undercarboxylated MGP in the circulation of PXE patients. Uitto et al. also reported the presence of mostly undercarboxylated MGP in the calcified vibrissae of the *Abcc6*^−/−^ mice [198]. At about the same time, a remarkably PXE-like phenotype was described in patients carrying mutations in the gamma-glutamyl carboxylase gene (*GGCX*) that encodes an enzyme essential for the carboxylation of MGP and OC as well as clotting factors [199]. Because vitamin K is an essential component in the post-translational carboxylation of glutamate residues (Glu) in these proteins, it was logically proposed that a vitamin K precursor or a conjugated form was the potential substrate(s) for ABCC6 and that insufficient carboxylation of MGP was the cause of abnormal calcification in PXE [200]. The fact that PXE patients have low serum levels of vitamin K [201], and that skin fibroblasts from patients present molecular signatures of impaired vitamin K-dependent carboxylation [202] gave further support to this notion. It was the first hypothesis that not only provided a strong and credible explanation to the calcification phenotype of PXE, but was also easily testable and offered the prospect of an affordable treatment. The PXE scientific community lost no time in developing competing studies using *Abcc6*^−/−^ mice to test various active forms of vitamin K, primarily K1 and K2 (i.e., phylloquinone or menaquinone), through dietary supplementation. In quick succession, three publications reported disappointing results, as none of the vitamin K forms tested at high or low concentrations stopped or reversed PXE mineralization [92,140,141]. Despite these negative results, interest in the relationship between vitamin K metabolism and abnormal calcification in PXE and GACI continues and several follow-up studies have since been published [115,116,142,143]. However, neither phylloquinone or menaquinone are considered to be viable treatment options for the time being.

#### 6.7.4. Bisphosphonate Treatment for PXE and GACI

Bisphosphonates are non-hydrolyzable analogs of PPi with two phosphonate (PO_3_) groups covalently linked to a central carbon (instead of an oxygen) and two side chains that determine chemical characteristics. There are two categories of bisphosphonates based on the presence of a non-nitrogenous or nitrogenous side chain. Because bisphosphonates mimic the structure of PPi, they have similar properties and can inhibit enzymes that utilize pyrophosphate. Bisphosphonates have been used clinically for decades in the treatment of osteoporosis and Paget’s disease of bone, as well as other applications related to mineralization.

The bisphosphonate etidronate has been used to treat GACI patients and early treatments improved GACI outcome [145]. Retrospective analyses have confirmed that survival beyond infancy is markedly improved with etidronate therapy [55,146]. However, this treatment seems to induce undesirable side-effects as a recent case report highlighted significant inhibition of skeletal calcification with paradoxical joint calcifications in a 7-year-old GACI patient [145]. Furthermore, studies on uremic rats or *Enpp1^−/−^* mice suggested that this bisphosphonate is incapable of preventing *de novo* calcification without alteration to bone structures [203,204].

For PXE, a first study reported the anti-calcification properties of zoledronate (nitrogenous) in an in vitro series of experiments with primary fibroblasts [147]. However, more elaborate studies with mouse models showed that only administration of etidronate (non-nitrogenous), but not alendronate (nitrogenous), prevented ectopic mineralization. However, etidronate treatment did not reverse existing mineralization [18,144,148]. The effect of etidronate was accompanied by alterations in the trabecular bone microarchitecture similar to what was described for *Enpp1^−/−^* mice. Although the results suggested that biphosphonates could be used as potential treatments for PXE, the high dosages and/or the bone alterations are potentially detrimental in the long term.

However, these animal studies and the fact that etidronate is an approved drug already tested for off-label applications against calcification in GACI and basal ganglia calcifications [205] were sufficiently convincing that a clinical trial termed Treatment of Ectopic Mineralization in PXE or TEMP was initiated in the Netherlands [126]. This investigation primarily tested effectiveness and safety of etidronate administration in PXE. The 12-month long investigation used a cyclical treatment of 20 mg/kg for 2 weeks every 12 weeks in a randomized, placebo-controlled phase I/II with 74 participants. The primary outcome was ectopic calcification as determined by ^18^fluoride positron emission tomography scans in femoral arterial tissues. Secondary outcomes were computed tomography arterial calcification and ophthalmological changes. Safety outcomes were bone density, serum calcium, and phosphate. The overall results were somewhat mixed, with PXE patients that received etidronate seeing reduced arterial calcification and subretinal neovascularization events. However, they did not experience lower femoral ^18^fluoride positron emission tomography activity, suggesting that active mineralization was still ongoing. Overall, no adverse effects from the treatment were reported.

Of note, another phase I/II clinical trial with a French cohort is in preparation and is called PyROphosPHate supplementation to fight ECtopIc calcification in PXE (PROPHECI, registration: https://scanr.enseignementsup-recherche.gouv.fr/project/PHRCN-PHRC-19-0402, accessed on 2 March 2021). This trial will use oral disodium PPi, but etidronate will be used as a control instead of a placebo.

#### 6.7.5. Pyrophosphate (PPi)

The idea of using PPi as a therapeutic for PXE and GACI came quickly after the discovery that ABCC6 modulates PPi production [8,9], though the idea of using PPi to prevent ectopic calcification was not new [206,207]. Considering the short half-life of PPi in plasma, several experimental approaches were devised to counteract calcification in PXE and GACI mouse models. Two groups proceeded to test different methodologies, with either a single daily bolus injection [18] or continuous delivery via drinking water [120]. The first study found that daily injections achieved near complete suppression of ectopic mineralization in several tissues despite a relatively poor bioavailability of about 0.5%. Importantly, and similar to other forms of treatments tested thus far, established mineralization could not be reversed [18].

Because daily injections are not necessarily practical for the lifelong treatment needed for PXE or GACI patients, oral administration was also evaluated [120]. Adding sodium PPi to drinking water sustainably raised plasma PPi in both *Abcc6*^−/−^ mice as well as in human volunteers, and significantly decreased calcification in both PXE and GACI mouse models. However, calcification inhibition was not complete. One of the remarkable discoveries of the latter study was that, contrary to previous beliefs [208], PPi is bioavailable when administered orally, although poorly (~0.1%), but is still effective against mineralization.

Furthermore, there has been a recent and inadvertent discovery that modulating PPi from dietary sources can also be effective at reducing calcification in *Abcc6*^−/−^ mice [19]. These findings were prompted by a routine change in the supplier of institutional rodent diet. The new chow was enriched in PPi (a fact unknown to the manufacturer and the research laboratory), leading to a doubling of plasma PPi and a halving of calcification in *Abcc6*^−/−^ mice. Dietary PPi is also readily absorbed in humans with a bioavailability comparable to that of drinking water (~0.1%), which suggests that dietary preference could contribute to the considerable and still unexplained phenotypic heterogeneity in PXE [19].

These recent studies unambiguously showed that PPi supplementation via oral delivery or even by injection are arguably the most promising and credible strategies for treating PXE (and GACI) patients. Indeed, PPi would be a simple and low-cost medication and it has a very safe profile. It is a nontoxic, physiological metabolite as per the World Health Organization (WHO). The US Food and Drug Administration (FDA) lists it as Generally Recognized As Safe and it is designated as food additive E450(a) in Europe. PPi is thus widely used in the food industry as a preservative in canned seafood, in baking soda, in cured meat, etc.… and is even present in toothpaste.

For these reasons, a phase II clinical trial using capsulized disodium PPi is currently being conducted (NCT04441671) while the PROPHECI clinical trial (see above) for PXE patients is currently in preparation and also proposes to test the oral delivery of encapsulated disodium PPi.

Of note, several pharmaceutical companies are now investing in the testing and manufacturing of new PPi formulations for the treatment of calcification in PXE, GACI, and other diseases.

#### 6.7.6. Phytic Acid (IP6)

Phytic acid (*myo*-inositol hexakisphosphate; IP6) is a phosphate ester of inositol. Its anti-calcification properties have been known for some time [209,210]. IP6 has poor bioavailability and pharmacokinetics, which has prompted the development of a series of derivatives [211]. These were tested in *Abcc6*^−/−^ mice [149]. Compound INS-3001 was found to be superior in calcification inhibition both in vitro and in vivo and displayed a better plasma half-life as compared with IP6 [211]. INS-3001 was administered to *Abcc6*^−/−^ mice before and after the onset of mineralization at various dosages via two methods, either microosmostic pumps for continuous delivery or sub-cutaneous bolus injections. With the highest dosages of 4 to 20 mg/kg/day, calcification inhibition in vibrissae was effective, though calcification reversal was not attained in the longest duration of the treatment (12 weeks). Although phytic acid (IP6) is a safe compound found in food such as plant seed and had the FDA designation Generally Recognized as Safe (GRAS), INS-3001 is a new product with good potential but it remains to be fully evaluated for clinical use.

#### 6.7.7. Sodium Thiosulfate

Sodium thiosulfate (Na_2_S_2_O_3_) is an industrial compound with a long clinical history [212] and it is commonly employed as a food preservative. It was originally used as an intravenous medication for metal poisoning [213]. It is now approved for the treatment of certain rare medical conditions notably calciphylaxis [214]. For this reason, intravenous sodium thiosulfate was recently administered to a single PXE patient with polygenic inheritance and severe early-onset manifestations [59]. This treatment achieved a remarkable regression of calcific stenosis in the coeliac and mesenteric arteries. However, significant side-effects resulted in discontinuation of the treatment and in the relapse of the symptoms. It is unclear if this treatment could be generalized to PXE and/or GACI patients but its potential use for the reversal of existing calcification should be explored, perhaps as a temporary measure to reduce existing calcification before a long-term inhibitory treatment can be applied.

## 7. Conclusions

We have learned much about ABCC6 [21] since its initial discovery 20 years ago [5,6,7], from its transcriptional regulation [215,216,217], expression profile [79,81,128], and molecular function and connections to an existing molecular pathway [8,9], to means and ways to rescue this transporter and treatments for PXE [18,19,92,98,99]. In two decades, PXE has gone from gene discovery to early clinical trials [126,218], a remarkable feat for rare disorders with significant morbidity but low mortality. At the time of this writing, PXE alone has been the subject of more than 2000 peer-reviewed publications (PubMed citations) as well as many other book chapters and reviews.

The study of PXE and GACI (and CALJA) has brought to light new fundamental knowledge about abnormal calcification and may have important implications for many other pathological contexts in humans. Indeed, the molecular pathway and mechanisms involved in PXE and the related diseases described above are likely to apply to common age-associated disorders such as chronic kidney insufficiency, diabetes, atherosclerosis, inflammatory skin diseases (sarcoidosis, systemic lupus erythematosus, scleroderma, and dermatomyositis, to name a few). Simple aging is also accompanied by soft tissue mineralization. Factors such as inflammation, infections, metabolic alterations, and genetics greatly influence and can exacerbate the mineralization process. When present, vascular calcification, an age-old condition [219], is predictive of worse clinical outcomes, with individual risk for cardiovascular mortality, or any cardiovascular event, dramatically increasing [220]. We estimate the frequency of heterozygous carriers of *ABCC6* mutations could be as high as 1/80 in the general population [86,221]. Thus, understanding how ABCC6 (and NPP1) influences the homeostasis of connective tissues may one day be used to enhance tissue repair and lessen mineralization-associated morbidity and mortality in the general population. This is particularly important in light of recent reports suggesting that three of the genes in the pathway shown in Figure 1 and Figure 3 that cause ectopic calcification in PXE, GACI, and CALJA [6,8,9,10,11,17,18,20] have also been shown to play a role in dyslipidemia and atherosclerosis [93,94,222,223,224,225].

Of all the treatment options explored above, none of them have focused and/or achieved the removal or reversal of existing calcification in PXE and GACI, save for one case of sodium thiosulfate usage. If past history has taught us anything, for diseases as complex as PXE and GACI, no single treatment will cover all aspects of these pathologies and there will be room for multiple forms of treatments for either disease, whether applied in combination or in single application.

What is next? The central role of ABCC6 in PXE, GACI, and DCC is now well established in humans [56] and animal models [112,226]. However, many aspects of the pathophysiology of ABCC6 dysfunction are still unexplained. The liver expression of ABCC6 is necessary but not sufficient for calcification inhibition [13,20]. The identification and contribution of peripheral tissues to calcification regulation remains unresolved. Plasma PPi levels fail to explain the wide range of calcification severity in humans [17] and mice [111] (*Cf* Figure 1) and the clinical relevance of potential modifier genes [109,110,147,150,154,155,156] is still unknown. Finally, what is (if any) the role of inflammation in the progression of PXE [159]? Answering some of these questions will not only inform the scientific community on these intriguing diseases but will ultimately help to refine and expand upon the various treatment options explored today.

## Figures and Tables

**Table 1:** Preclinical studies and early clinical trials for PXE (and GACI).

Treatment/Therapy	Rationale/Target	Diagnosis	References
Correction, replacement, or inhibition of dysfunctional genes/proteins			
PTC-124 (Ataluren or Translarna)	Allows read-through of PTC codons, targets nonsense mutations	PXE	[131]
4-Phenylbutyrate (4-PBA)	Corrects missense mutations allowing for correct cellular localization	PXE	[80,99,117]
Rh-NPP1-Fc	Replacement for ENPP1	GACI	[89,132,133]
SBI-425 (TNAP inhibitor)	Inhibits the enzymatic activity of TNAP	PXE	[20,127]
Fetuin-A	Glycoprotein that forms complexes with calcium and phosphate ions, acts as an inhibitor of ectopic calcification	PXE	[134,135]
Adenovirus with ABCC6 cDNA	Transiently express ABCC6 in the liver	PXE	[136]
Bevacizumab (Anti-VEGF) †	Anti-angiogenic therapy; Preserves ocular function in advanced and early disease stages	PXE	[24]
Supplementation therapies for direct inhibition of calcification			
Sevelamer hydrochloride (Renagel)	Phosphate binder	PXE	[130,137]
Magnesium	Inhibits the formation of apatite	PXE	[129,138,139]
Vitamin K (phylloquinone/menaquinone)	Correct for insufficient carboxylation of matrix gla protein (MGP)	PXE	[92,115,116,140,141,142,143]
Bisphosphonate (etidronate *, zoledronate)	Non-hydrolyzable analog of PPi, inhibits enzymes that utilize pyrophosphate	PXE and GACI	[18,55,126,144,145,146,147,148]
Pyrophosphate *	Potent inhibitor of calcification, ABCC6 modulates PPi production	PXE	[18,19,120], PROPHECI
INS-3001 (IP6 derivative)	Known inhibitor of calcification	PXE	[149]
Sodium thiosulfate	Approved for calciphlaxis	PXE	[59]

* Denotes treatments undergoing clinical trials, † currently in use in PXE patients. PXE, pseudoxanthoma elasticum; GACI, generalized arterial calcification of infancy; MGP, matrix gla protein; PPi, pyrophosphate.

## Data Availability

Not applicable.

## References

[1] He K., Sawczyk M., Liu C., Yuan Y., Song B., Deivanayagam R., Nie A., Hu X., Dravid V.P., Lu J. (2020). Revealing nanoscale mineralization pathways of hydroxyapatite using in situ liquid cell transmission electron microscopy. Sci. Adv..

[2] Atzeni F., Sarzi-Puttini P., Bevilacqua M. (2006). Calcium deposition and associated chronic diseases (atherosclerosis, diffuse idiopathic skeletal hyperostosis, and others). Rheum. Dis. Clin. N. Am..

[3] Rutsch F., Nitschke Y., Terkeltaub R. (2011). Genetics in arterial calcification: Pieces of a puzzle and cogs in a wheel. Circ. Res..

[4] Ruiz J.L., Hutcheson J.D., Aikawa E. (2015). Cardiovascular calcification: Current controversies and novel concepts. Cardiovasc. Pathol..

[5] Bergen A.A., Plomp A.S., Schuurman E.J., Terry S.F., Breuning M.H., Dauwerse H.G., Swart J., Kool M., van Soest S., Baas F. (2000). Mutations in ABCC6 cause pseudoxanthoma elasticum. Nat. Genet..

[6] Le Saux O., Urban Z., Tschuch C., Csiszar K., Bacchelli B., Quaglino D., Pasquali-Ronchetti I., Pope F.M., Richards A., Terry S.F. (2000). Mutations in a gene encoding an ABC transporter cause pseudoxanthoma elasticum. Nat. Genet..

[7] Ringpfeil F., Lebwohl M.G., Christiano A.M., Uitto J. (2000). Pseudoxanthoma elasticum: Mutations in the MRP6 gene encoding a transmembrane ATP-binding cassette (ABC) transporter. Proc. Natl. Acad. Sci. USA.

[8] Jansen R.S., Küçükosmanoglu A., de Haas M., Sapthu S., Otero J.A., Hegman I.E.M., Bergen A.A.B., Gorgels T.G.M.F., Borst P., van de Wetering K. (2013). ABCC6 prevents ectopic mineralization seen in pseudoxanthoma elasticum by inducing cellular nucleotide release. Proc. Natl. Acad. Sci. USA.

[9] Jansen R.S., Duijst S., Mahakena S., Sommer D., Szeri F., Váradi A., Plomp A.S., Bergen A.A.B., Elferink R.P.J.O., Borst P. (2014). ABCC6–Mediated ATP Secretion by the Liver Is the Main Source of the Mineralization Inhibitor Inorganic Pyrophosphate in the Systemic Circulation—Brief Report. Arter. Thromb. Vasc. Biol..

[10] Markello T.C., Pak L.K., St. Hilaire C., Dorward H., Ziegler S.G., Chen M.Y., Chaganti K., Nussbaum R.L., Boehm M., Gahl W.A. (2011). Vascular pathology of medial arterial calcifications in NT5E deficiency: Implications for the role of adenosine in pseudoxanthoma elasticum. Mol. Genet. Metab..

[11] Miglionico R., Armentano M.F., Carmosino M., Salvia A.M., Cuviello F., Bisaccia F., Ostuni A. (2014). Dysregulation of gene expression in ABCC6 knockdown HepG2 cells. Cell. Mol. Biol. Lett..

[12] Aherrahrou Z., Doehring L.C., Ehlers E.-M., Liptau H., Depping R., Linsel-Nitschke P., Kaczmarek P.M., Erdmann J., Schunkert H. (2008). An Alternative Splice Variant in Abcc6, the Gene Causing Dystrophic Calcification, Leads to Protein Deficiency in C3H/He Mice. J. Biol. Chem..

[13] Brampton C., Aherrahrou Z., Chen L.-H., Martin L., Bergen A.A., Gorgels T.G., Erdfdi J., Schunkert H., Szabó Z., Váradi A. (2014). The Level of Hepatic ABCC6 Expression Determines the Severity of Calcification after Cardiac Injury. Am. J. Pathol..

[14] Meng H., Vera I., Che N., Wang X., Wang S.S., Ingram-Drake L., Schadt E.E., Drake T.A., Lusis A.J. (2007). Identification of Abcc6 as the major causal gene for dystrophic cardiac calcification in mice through inte-grative genomics. Proc. Natl. Acad. Sci. USA.

[15] Kalal I.G., Seetha D., Panda A., Nitschke Y., Rutsch F. (2012). Molecular diagnosis of generalized arterial calcification of infancy (GACI). J. Cardiovasc. Dis. Res..

[16] Le Boulanger G., Labreze C., Croue A., Schurgers L.J., Chassaing N., Wittkampf T., Rutsch F., Martin L. (2010). An unusual severe vascular case of pseudoxanthoma elasticum presenting as generalized arterial calcifica-tion of infancy. Am. J. Med. Genet. A.

[17] Nitschke Y., Baujat G., Botschen U., Wittkampf T., du Moulin M., Stella J., le Merrer M., Guest G., Lambot K., Tazarourte-Pinturier M.-F. (2012). Generalized Arterial Calcification of Infancy and Pseudoxanthoma Elasticum Can Be Caused by Mutations in Either ENPP1 or ABCC6. Am. J. Hum. Genet..

[18] Pomozi V., Brampton C., van de Wetering K., Zoll J., Calio B., Pham K., Owens J.B., Marh J., Moisyadi S., Váradi A. (2017). Pyrophosphate Supplementation Prevents Chronic and Acute Calcification in ABCC6-Deficient Mice. Am. J. Pathol..

[19] Pomozi V., Julian C.B., Zoll J., Pham K., Kuo S., Tőkési N., Martin L., Váradi A., le Saux O. (2019). Dietary Pyrophosphate Modulates Calcification in a Mouse Model of Pseudoxanthoma Elasticum: Implication for Treatment of Patients. J. Investig. Dermatol..

[20] Ziegler S.G., Ferreira C.R., Macfarlane E.G., Riddle R.C., Tomlinson R.E., Chew E.Y., Martin L., Ma C.-T., Sergienko E., Pinkerton A.B. (2017). Ectopic calcification in pseudoxanthoma elasticum responds to inhibition of tissue-nonspecific alkaline phosphatase. Sci. Transl. Med..

[21] Borst P., Váradi A., van de Wetering K. (2019). PXE, a Mysterious Inborn Error Clarified. Trends Biochem. Sci..

[22] Ali S.A., Ng C., Votava-Smith J.K., Randolph L.M., Pitukcheewanont P. (2018). Bisphosphonate therapy in an infant with generalized arterial calcification with an ABCC6 mutation. Osteoporos. Int..

[23] Edouard T., Chabot G., Miro J., Buhas D.C., Nitschke Y., Lapierre C., Rutsch F., Alos N. (2011). Efficacy and safety of 2-year etidronate treatment in a child with generalized arterial calcification of infancy. Eur. J. Nucl. Med. Mol. Imaging.

[24] Finger R.P., Issa P.C., Schmitz-Valckenberg S., Holz F.G., Scholl H.N. (2011). Long-Term Effectiveness of Intravitreal Bevacizumab for Choroidal Neovascularization Secondary to Angioid Streaks in Pseudoxanthoma Elasticum. Retina.

[25] Uitto J., Shamban A. (1987). Heritable Skin Diseases with Molecular Defects in Collagen or Elastin. Dermatol. Clin..

[26] Balzer F. (1884). Recherches sur les caractères anatomiques du xanthelasma. Arch. Physiol..

[27] Chauffard M.A. (1889). Xanthélasma disséminé et symétrique et sans insuffisance hépatique. Bull. Soc. Med. Paris.

[28] Rigal D. (1881). Observation pour servir à l’histoire de la chéloide diffuse xanthélasmique. Ann. Dermatol. Syphilol..

[29] Darier J. (1896). Pseudo-Xanthome Elastique.

[30] Neldner K.H. (1988). Pseudoxanthoma Elasticum. Int. J. Dermatol..

[31] Truter S., Rosenbaum-Fiedler J., Sapadin A., Lebwohl M. (1996). Calcification of elastic fibers in pseudoxan-thoma elasticum. Mt. Sinai. J. Med..

[32] Uitto J., Boyd C.D., Lebwohl M.G., Moshell A.N., Rosenbloom J., Terry S. (1998). International Centennial Meeting on Pseudoxanthoma Elasticum: Progress in PXE Research. J. Investig. Dermatol..

[33] Weenink A.C., Dijkman G., de Meijer P.H. (1996). Pseudoxanthoma elasticum and its complications: Two case reports. Neth. J. Med..

[34] Campens L., Vanakker O.M., Trachet B., Segers P., Leroy B.P., de Zaeytijd J., Voet D., de Paepe A., de Backer T., de Backer J. (2013). Characterization of Cardiovascular Involvement in Pseudoxanthoma Elasticum Families. Arter. Thromb. Vasc. Biol..

[35] Germain D.P., Boutouyrie P., Laloux B., Laurent S. (2003). Arterial Remodeling and Stiffness in Patients with Pseudoxanthoma Elasticum. Arter. Thromb. Vasc. Biol..

[36] Kornet L., Bergen A.A., Hoeks A.P., Cleutjens J.P., Oostra R.-J., Daemen M.J., van Soest S., Reneman R.S. (2004). In patients with pseudoxanthoma elasticum a thicker and more elastic carotid artery is associated with elastin fragmentation and proteoglycans accumulation. Ultrasound Med. Biol..

[37] Bertulezzi G., Paris R., Moroni M., Porta C., Nastasi G., Amadeo A. (1998). Atrial septal aneurysm in a patient with pseudoxanthoma elasticum. Acta Cardiol..

[38] Prunier F., Terrien G., le Corre Y., Apana A.L.Y., Bière L., Kauffenstein G., Furber A., Bergen A.A.B., Gorgels T.G.M.F., Le Saux O. (2013). Pseudoxanthoma Elasticum: Cardiac Findings in Patients and Abcc6-Deficient Mouse Model. PLoS ONE.

[39] Eddy D.D., Farber E.M. (1962). Pseudoxathoam elasticum. Internal manifestatons: A report of cases and a review of the literature. Arch. Dermatol..

[40] Kauffenstein G., Pizard A., le Corre Y., Vessières E., Grimaud L., Toutain B., Labat C., Mauras Y., Gorgels T.G., Bergen A.A. (2014). Disseminated arterial calcification and enhanced myogenic response are associated with abcc6 deficiency in a mouse model of pseudoxanthoma elasticum. Arter. Thromb. Vasc. Biol..

[41] Fabre B., Bayle P., Bazex J., Durand D., Lamant L., Chassaing N. (2005). Pseudoxanthoma elasticum and nephrolithiasis. J. Eur. Acad. Dermatol. Venereol..

[42] Mallette L.E., Mechanick J.I. (1987). Heritable syndrome of pseudoxanthoma elasticum with abnormal phosphorus and vitamin D metabolism. Am. J. Med..

[43] Seeger H., Mohebbi N. (2016). Pseudoxanthoma elasticum and nephrocalcinosis. Kidney Int..

[44] Legrand A., Cornez L., Samkari W., Mazzella J.-M., Venisse A., Boccio V., Auribault K., Keren B., Benistan K., Germain D.P. (2017). Mutation spectrum in the ABCC6 gene and genotype–phenotype correlations in a French cohort with pseudoxanthoma elasticum. Genet. Med..

[45] Letavernier E., Bouderlique E., Zaworski J., Martin L., Daudon M. (2019). Pseudoxanthoma Elasticum, Kidney Stones and Pyrophosphate: From a Rare Disease to Urolithiasis and Vascular Calcifications. Int. J. Mol. Sci..

[46] Letavernier E., Kauffenstein G., Huguet L., Navasiolava N., Bouderlique E., Tang E., Delaitre L., Bazin D., de Frutos M., Gay C. (2018). ABCC6 Deficiency Promotes Development of Randall Plaque. J. Am. Soc. Nephrol..

[47] Gheduzzi D., Sammarco R., Quaglino D., Bercovitch L., Terry S., Taylor W., Ronchetti I.P. (2003). Extracutaneous ultrastructural alterations in pseudoxanthoma elasticum. Ultrastruct. Pathol..

[48] Lebwohl M., Halperin J., Phelps R.G. (1993). Brief report: Occult pseudoxanthoma elasticum in patients with premature cardiovascular disease. N. Engl. J. Med..

[49] Rutsch F., Vaingankar S., Johnson K., Goldfine I., Maddux B., Schauerte P., Kalhoff H., Sano K., Boisvert W.A., Superti-Furga A. (2001). PC-1 Nucleoside Triphosphate Pyrophosphohydrolase Deficiency in Idiopathic Infantile Arterial Calcification. Am. J. Pathol..

[50] Moran J.J. (1975). Idiopathic arterial calcification of infancy: A clinicopathologic study. Pathol. Annu..

[51] Morton R. (1978). Idiopathic arterial calcification in infancy. Histopathology.

[52] Ramjan K.A., Roscioli T., Rutsch F., Sillence D., Munns C.F. (2009). Generalized arterial calcification of infancy: Treatment with bisphosphonates. Nat. Clin. Pract. Endocrinol. Metab..

[53] Rutsch F., Boyer P., Nitschke Y., Ruf N., Lorenz-Depierieux B., Wittkampf T., Weissen-Plenz G., Fischer R.J., Mughal Z., Gregory J.W. (2008). Hypophosphatemia, hyperphosphaturia, and bisphosphonate treatment are associated with survival beyond infancy in generalized arterial calcification of infancy. Circ. Cardiovasc. Genet..

[54] Lorenz-Depiereux B., Schnabel D., Tiosano D., Hausler G., Strom T.M. (2010). Loss-of-function ENPP1 mutations cause both generalized arterial calcification of infancy and autosomal-recessive hypophosphatemic rickets. Am. J. Hum. Genet..

[55] Rutsch F., Ruf N., Vaingankar S., Toliat M.R., Suk A., Hohne W., Schauer G., Lehmann M., Roscioli T., Schnabel D. (2003). Mutations in ENPP1 are associated with ’idiopathic’ infantile arterial calcification. Nat. Genet..

[56] Le Saux O., Martin L., Aherrahrou Z., Leftheriotis G., Varadi A., Brampton C.N. (2012). The molecular and physiological roles of ABCC6: More than meets the eye. Front. Genet..

[57] Nitschke Y., Rutsch F. (2012). Generalized arterial calcification of infancy and pseudoxanthoma elasticum: Two sides of the same coin. Front. Genet..

[58] Li Q., Grange D.K., Armstrong N.L., Whelan A.J., Hurley M.Y., Rishavy M.A., Hallgren K.W., Berkner K.L., Schurgers L.J., Jiang Q. (2009). Mutations in the GGCX and ABCC6 Genes in a Family with Pseudoxanthoma Elasticum-Like Phenotypes. J. Investig. Dermatol..

[59] Omarjee L., Nitschke Y., Verschuere S., Bourrat E., Vignon M., Navasiolava N., Leftheriotis G., Kauffenstein G., Rutsch F., Vanakker O. (2019). Severe early-onset manifestations of pseudoxanthoma elasticum resulting from the cumulative effects of several deleterious mutations in ENPP1, ABCC6 and HBB: Transient improvement in ectopic calcification with sodium thiosulfate. Br. J. Dermatol..

[60] Bentley-Phillips B. (1985). Pseudoxanthoma elasticum-like skin changes induced by penicillamine. J. R. Soc. Med..

[61] Bolognia J.L., Braverman I. (1992). Pseudoxanthoma-elasticum-like Skin Changes Induced by Penicillamine. Dermatology.

[62] Coatesworth A.P., Darnton S.J., Green R.M., Cayton R.M., Antonakopoulos G.N. (1998). A case of systemic pseudo-pseudoxanthoma elasticum with diverse symptomatology caused by long-term penicillamine use. J. Clin. Pathol..

[63] Dhurat R., Nayak C., Pereira R., Kagne R., Khatu S. (2011). Penicillamine-induced elastosis perforans serpiginosa with abnormal “lumpy-bumpy” elastic fibers in lesional and non-lesional skin. Indian J. Dermatol. Venereol. Leprol..

[64] Aessopos A., Farmakis D., Loukopoulos D. (2002). Elastic tissue abnormalities resembling pseudoxanthoma elasticum in beta thalassemia and the sickling syndromes. Blood.

[65] Baccarani-Contri M., Bacchelli B., Boraldi F., Quaglino D., Taparelli F., Carnevali E., Francomano M.A., Seidenari S., Bettoli V., de Sanctis V. (2001). Characterization of pseudoxanthoma elasticum-like lesions in the skin of patients with beta-thalassemia. J. Am. Acad. Dermatol..

[66] Cianciulli P., Sorrentino F., Maffei L., Amadori S., Cappabianca M.P., Foglietta E., Carnevali E., Pasquali-Ronchetti I. (2002). Cardiovascular involvement in thalassaemic patients with pseudoxanthoma elasticum-like skin lesions: A long-term follow-up study. Eur. J. Clin. Investig..

[67] Farmakis D., Moyssakis I., Perakis A., Rombos Y., Deftereos S., Giakoumis A., Polymeropoulos E., Aessopos A. (2003). Unstable angina associated with coronary arterial calcification in a thalassemia intermedia patient with a pseudoxanthoma elasticum-like syndrome. Eur. J. Haematol..

[68] Farmakis D., Vesleme V., Papadogianni A., Tsaftaridis P., Kapralos P., Aessopos A. (2004). Aneurysmatic dilatation of ascending aorta in a patient with beta-thalassemia and a pseudoxanthoma elasticum-like syndrome. Ann. Hematol..

[69] Hamlin N., Beck K., Bacchelli B., Cianciulli P., Pasquali-Ronchetti I., le Saux O. (2003). Acquired Pseudoxanthoma elasticum-like syndrome in beta-thalassaemia patients. Br. J. Haematol..

[70] Klein I., Sarkadi B., Váradi A. (1999). An inventory of the human ABC proteins. Biochim. Biophys. Acta Biomembr..

[71] Stefková J., Poledne R., Hubácek J.A. (2004). ATP-binding cassette (ABC) transporters in human metabolism and diseases. Physiol. Res..

[72] Dawson R.J., Locher K.P. (2007). Structure of the multidrug ABC transporter Sav1866 from *Staphylococcus aureus* in complex with AMP-PNP. FEBS Lett..

[73] Fülöp K., Barna L., Symmons O., Závodszky P., Váradi A. (2009). Clustering of disease-causing mutations on the domain–domain interfaces of ABCC6. Biochem. Biophys. Res. Commun..

[74] Madon J., Hagenbuch B., Landmann L., Meier P.J., Stieger B. (2000). Transport Function and Hepatocellular Localization of mrp6 in Rat Liver. Mol. Pharmacol..

[75] Cai J., Daoud R., Alqawi O., Georges E., Pelletier J., Gros P. (2002). Nucleotide binding and nucleotide hydrolysis properties of the ABC transporter MRP6 (ABCC6). Biochemistry.

[76] Ilias A., Urban Z., Seidl T.L., le Saux O., Sinko E., Boyd C.D., Sarkadi B., Varadi A. (2002). Loss of ATP-dependent transport activity in pseudoxanthoma elasticum-associated mutants of human ABCC6 (MRP6). J. Biol. Chem..

[77] Hirohashi T., Suzuki H., Sugiyama Y. (1999). Characterization of the transport properties of cloned rat multi-drug resistance-associated protein 3 (MRP3). J. Biol. Chem..

[78] Belinsky M.G., Chen Z.-S., Shchaveleva I., Zeng H., Kruh G.D. (2002). Characterization of the drug resistance and transport properties of multidrug resistance protein 6 (MRP6, ABCC6). Cancer Res..

[79] Beck K., Hayashi K., Nishiguchi B., le Saux O., Hayashi M., Boyd C.D. (2003). The Distribution of Abcc6 in Normal Mouse Tissues Suggests Multiple Functions for this ABC Transporter. J. Histochem. Cytochem..

[80] Le Saux O., Fülöp K., Yamaguchi Y., Iliás A., Szabó Z., Brampton C.N., Pomozi V., Huszár K., Arányi T., Váradi A. (2011). Expression and In Vivo Rescue of Human ABCC6 Disease-Causing Mutants in Mouse Liver. PLoS ONE.

[81] Beck K., Hayashi K., Dang K., Hayashi M., Boyd C.D. (2005). Analysis of ABCC6 (MRP6) in normal human tissues. Histochem. Cell Biol..

[82] Sinkó E., Iliás A., Ujhelly O., Homolya L., Scheffer G.L., Bergen A.A.B., Sarkadi B., Váradi A. (2003). Subcellular localization and N-glycosylation of human ABCC6, expressed in MDCKII cells. Biochem. Biophys. Res. Commun..

[83] Martin L.J., Lau E., Singh H., Vergnes L., Tarling E.J., Mehrabian M., Mungrue I., Xiao S., Shih D., Castellani L. (2012). ABCC6 localizes to the mitochondria-associated membrane. Circ. Res..

[84] Kato K., Nishimasu H., Okudaira S., Mihara E., Ishitani R., Takagi J., Aoki J., Nureki O. (2012). Crystal structure of Enpp1, an extracellular glycoprotein involved in bone mineralization and insulin signaling. Proc. Natl. Acad. Sci. USA.

[85] Roberts F., Zhu D., Farquharson C., Macrae V.E. (2019). ENPP1 in the Regulation of Mineralization and Beyond. Trends Biochem. Sci..

[86] Chassaing N., Martin L., Calvas P., le Bert M., Hovnanian A. (2005). Pseudoxanthoma elasticum: A clinical, pathophysiological and genetic update including 11 novel ABCC6 mutations. J. Med Genet..

[87] Le Saux O., Beck K., Sachsinger C., Silvestri C., Treiber C., Goring H.H., Johnson E.W., de Paepe A., Pope F.M., Pasquali-Ronchetti I. (2001). A spectrum of abcc6 mutations is responsible for pseudoxanthoma elasticum. Am. J. Hum. Genet..

[88] Pfendner E.G., Vanakker O.M., Terry P.F., Vourthis S., McAndrew E.P., McClain M.R., Fratta S., Marais A.-S., Hariri S., Coucke P.J. (2007). Mutation detection in the ABCC6 gene and genotype phenotype analysis in a large international case series affected by pseudoxanthoma elasticum. J. Med Genet..

[89] Nitschke Y., Yan Y., Buers I., Kintziger K., Askew K., Rutsch F. (2018). ENPP1-Fc prevents neointima formation in generalized arterial calcification of infancy through the generation of AMP. Exp. Mol. Med..

[90] Gorgels T.G., Hu X., Scheffer G.L., van der Wal A.C., Toonstra J., de Jong P.T., van Kuppevelt T.H., Levelt C.N., de Wolf A., Loves W.J. (2005). Disruption of Abcc6 in the mouse: Novel insight in the pathogenesis of pseudoxanthoma elasticum. Hum. Mol. Genet..

[91] Klement J.F., Matsuzaki Y., Jiang Q.-J., Terlizzi J., Choi H.Y., Fujimoto N., Li K., Pulkkinen L., Birk D.E., Sundberg J.P. (2005). Targeted Ablation of the Abcc6 Gene Results in Ectopic Mineralization of Connective Tissues. Mol. Cell. Biol..

[92] Brampton C., Yamaguchi Y., Vanakker O., van Laer L., Chen L.-H., Thakore M., de Paepe A., Pomozi V., Szabó P.T., Martin L. (2011). Vitamin K does not prevent soft tissue mineralization in a mouse model of pseudoxanthoma elasticum. Cell Cycle.

[93] Brampton C., Pomozi V., Chen L.-H., Apana A., McCurdy S., Zoll J., Boisvert W.A., Lambert G., Henrion D., Blanchard S. (2021). ABCC6 deficiency promotes dyslipidemia and atherosclerosis. Sci. Rep..

[94] Ibold B., Tiemann J., Faust I., Ceglarek U., Dittrich J., Gorgels T.G.M.F., Bergen A.A.B., Vanakker O., van Gils M., Knabbe C. (2021). Genetic deletion of Abcc6 disturbs cholesterol homeostasis in mice. Sci. Rep..

[95] Jiang Q., Endo M., Dibra F., Wang K., Uitto J. (2009). Pseudoxanthoma Elasticum Is a Metabolic Disease. J. Investig. Dermatol..

[96] Jiang Q., Oldenburg R., Otsuru S., Grand-Pierre A.E., Horwitz E.M., Uitto J. (2010). Parabiotic heterogenetic pairing of Abcc6−/−/Rag1−/− mice and their wild-type counterparts halts ectopic mineralization in a murine model of pseudoxanthoma elasticum. Am. J. Pathol..

[97] Le Saux O., Bunda S., van Wart C.M., Douet V., Got L., Martin L., Hinek A. (2006). Serum Factors from Pseudoxanthoma Elasticum Patients Alter Elastic Fiber Formation In Vitro. J. Investig. Dermatol..

[98] Li Q., Sundberg J.P., Levine M.A., Terry S.F., Uitto J. (2015). The effects of bisphosphonates on ectopic soft tissue mineralization caused by mutations in the ABCC6 gene. Cell Cycle.

[99] Pomozi V., Brampton C., Szeri F., Dedinszki D., Kozak E., van de Wetering K., Hopkins H., Martin L., Varadi A., le Saux O. (2017). Functional Rescue of ABCC6 Deficiency by 4-Phenylbutyrate Therapy Reduces Dystrophic Calcification in Abcc6(−/−) Mice. J. Investig. Dermatol..

[100] Doehring L.C., Kaczmarek P.M., Ehlers E.-M., Mayer B., Erdmann J., Schunkert H., Aherrahrou Z. (2006). Arterial calcification in mice after freeze-thaw injury. Ann. Anat. Anat. Anz..

[101] Eaton G.J., Custer R.P., Johnson F.N., Stabenow K.T. (1978). Dystrophic cardiac calcinosis in mice: Genetic, hormonal, and dietary influences. Am. J. Pathol..

[102] Everitt J.I., Olson L.M., Mangum J.B., Visek W.J. (1988). High mortality with severe dystrophic cardiac calcinosis in C3H/OUJ mice fed high fat purified diets. Vet. Pathol..

[103] Aherrahrou Z., Axtner S.B., Kaczmarek P.M., Jurat A., Korff S., Doehring L.C., Weichenhan D., Katus H.A., Ivandic B.T. (2004). A locus on chromosome 7 determines dramatic up-regulation of osteopontin in dystrophic cardiac calcification in mice. Am. J. Pathol..

[104] Ivandic B.T., Utz H.F., Kaczmarek P.M., Aherrahrou Z., Axtner S.B., Klepsch C., Lusis A.J., Katus H.A. (2001). New Dyscalc loci for myocardial cell necrosis and calcification (dystrophic cardiac calcinosis) in mice. Physiol. Genom..

[105] Brunnert S.R. (1997). Morphologic response of myocardium to freeze-thaw injury in mouse strains with dystrophic cardiac calcification. Lab. Anim. Sci..

[106] Smolen K.K., Gelinas L., Franzen L., Dobson S., Dawar M., Ogilvie G., Krajden M., Fortuno E.S., Kollmann T.R. (2012). Age of recipient and number of doses differentially impact human B and T cell immune memory responses to HPV vaccination. Vaccine.

[107] Li Q., Berndt A., Guo H., Sundberg J.P., Uitto J. (2012). A Novel Animal Model for Pseudoxanthoma Elasticum: The KK/HlJ Mouse. Am. J. Pathol..

[108] Berndt A., Sundberg B.A., Silva K.A., Kennedy V.E., Richardson M.A., Li Q., Bronson R.T., Uitto J., Sundberg J.P. (2013). Phenotypic Characterization of the KK/HlJ Inbred Mouse Strain. Veter. Pathol..

[109] De Vilder E.Y., Hosen M.J., Martin L., de Zaeytijd J., Leroy B.P., Ebran J., Coucke P.J., de Paepe A., Vanakker O.M. (2020). VEGFA variants as prognostic markers for the retinopathy in pseudoxanthoma elasticum. Clin. Genet..

[110] Luo H., Faghankhani M., Cao Y., Uitto J., Li Q. (2020). Molecular Genetics and Modifier Genes in Pseudoxanthoma Elasticum, a Heritable Multisystem Ectopic Mineralization Disorder. J. Investig. Dermatol..

[111] Le Corre Y., le Saux O., Froeliger F., Libouban H., Kauffenstein G., Willoteaux S., Leftheriotis G., Martin L. (2012). Quantification of the calcification phenotype of abcc6-deficient mice with microcomputed tomography. Am. J. Pathol..

[112] Li Q., Kingman J., van de Wetering K., Tannouri S., Sundberg J.P., Uitto J. (2017). Abcc6 Knockout Rat Model Highlights the Role of Liver in PPi Homeostasis in Pseudoxanthoma Elasticum. J. Investig. Dermatol..

[113] Li Q., Sadowski S., Frank M.M., Chai C., Váradi A., Ho S.-Y., Lou H., Dean M., Thisse C., Thisse B. (2010). The abcc6a Gene Expression Is Required for Normal Zebrafish Development. J. Investig. Dermatol..

[114] Van Gils M., Willaert A., de Vilder E., Coucke P., Vanakker O. (2018). Generation and Validation of a Complete Knockout Model of abcc6a in Zebrafish. J. Investig. Dermatol..

[115] Sun J., She P., Liu X., Gao B., Jin D., Zhong T.P. (2020). Disruption of Abcc6 Transporter in Zebrafish Causes Ocular Calcification and Cardiac Fibrosis. Int. J. Mol. Sci..

[116] Mackay E.W., Apschner A., Schulte-Merker S. (2015). Vitamin K reduces hypermineralisation in zebrafish models of PXE and GACI. Development.

[117] Pomozi V., Brampton C., Fülöp K., Chen L.-H., Apana A., Li Q., Uitto J., le Saux O., Váradi A. (2014). Analysis of Pseudoxanthoma Elasticum–Causing Missense Mutants of ABCC6 In Vivo; Pharmacological Correction of the Mislocalized Proteins. J. Investig. Dermatol..

[118] Okawa A., Nakamura I., Goto S., Moriya H., Nakamura Y., Ikegawa S. (1998). Mutation in Npps in a mouse model of ossification of the posterior longitudinal ligament of the spine. Nat. Genet..

[119] Li Q., Guo H., Chou D.W., Berndt A., Sundberg J.P., Uitto J. (2013). Mutant Enpp1asj mice as a model for generalized arterial calcification of infancy. Dis. Model. Mech..

[120] Dedinszki D., Szeri F., Kozak E., Pomozi V., Tokesi N., Mezei T.R., Merczel K., Letavernier E., Tang E., Le Saux O. (2017). Oral administration of pyrophosphate inhibits con-nective tissue calcification. EMBO Mol. Med..

[121] Huesa C., Zhu D., Glover J.D., Ferron M., Karsenty G., Milne E.M., Millan J.L., Ahmed S.F., Farquharson C., Morton N.M. (2014). Deficiency of the bone mineralization inhibitor NPP1 protects mice against obesity and diabetes. Dis. Model. Mech..

[122] Mackenzie N., Huesa C., Rutsch F., MacRae V. (2012). New insights into NPP1 function: Lessons from clinical and animal studies. Bone.

[123] Apschner A., Huitema L.F., Ponsioen B., Peterson-Maduro J., Schulte-Merker S. (2014). Zebrafish enpp1 mutants exhibit pathological mineralization, mimicking features of generalized arterial calcification of in-fancy (GACI) and pseudoxanthoma elasticum (PXE). Dis. Model Mech..

[124] Kauffenstein G., Yegutkin G.G., Khiati S., Pomozi V., le Saux O., Leftheriotis G., Lenaers G., Henrion D., Martin L. (2018). Alteration of Extracellular Nucleotide Metabolism in Pseudoxanthoma Elasticum. J. Investig. Dermatol..

[125] Uitto J., Li Q., van de Wetering K., Váradi A., Terry S.F. (2017). Insights into Pathomechanisms and Treatment Development in Heritable Ectopic Mineralization Disorders: Summary of the PXE International Biennial Research Symposium-2016. J. Investig. Dermatol..

[126] Kranenburg G., de Jong P.A., Bartstra J.W., Lagerweij S.J., Lam M.G., Norel J.O.-V., Risseeuw S., van Leeuwen R., Imhof S.M., Verhaar H.J. (2018). Etidronate for Prevention of Ectopic Mineralization in Patients with Pseudoxanthoma Elasticum. J. Am. Coll. Cardiol..

[127] Li Q., Huang J., Pinkerton A.B., Millan J.L., van Zelst B.D., Levine M.A., Sundberg J.P., Uitto J. (2019). Inhibition of Tissue-Nonspecific Alkaline Phosphatase Attenuates Ectopic Mineralization in the Abcc6 (−/−) Mouse Model of PXE but Not in the Enpp1 Mutant Mouse Models of GACI. J. Investig. Dermatol..

[128] Pomozi V., le Saux O., Brampton C., Apana A., Iliás A., Szeri F., Martin L., Monostory K., Paku S., Sarkadi B. (2013). ABCC6 Is a Basolateral Plasma Membrane Protein. Circ. Res..

[129] Rose S., On S.J., Fuchs W., Chen C., Phelps R., Kornreich D., Haddican M., Singer G., Wong V., Baum D. (2019). Magnesium Supplementation in the Treatment of Pseudoxanthoma Elasticum (PXE): A randomized trial. J. Am. Acad. Dermatol..

[130] Yoo J.Y., Blum R.R., Singer G.K., Stern D.K., Emanuel P.O., Fuchs W., Phelps R.G., Terry S.F., Lebwohl M.G. (2011). A randomized controlled trial of oral phosphate binders in the treatment of pseudoxanthoma elasticum. J. Am. Acad. Dermatol..

[131] Zhou Y., Jiang Q., Takahagi S., Shao C., Uitto J., Takahagi S. (2013). Premature termination codon read-through in the ABCC6 gene: Potential treatment for pseudoxanthoma elasticum. J. Investig. Dermatol..

[132] Albright R.A., Stabach P., Cao W., Kavanagh D., Mullen I., Braddock A.A., Covo M.S., Tehan M., Yang G., Cheng Z. (2015). ENPP1-Fc prevents mortality and vascular calcifications in rodent model of generalized arterial calcification of infancy. Nat. Commun..

[133] Khan T., Sinkevicius K.W., Vong S., Avakian A., Leavitt M.C., Malanson H., Marozsan A., Askew K.L. (2018). ENPP1 enzyme replacement therapy improves blood pressure and cardiovascular function in a mouse model of generalized arterial calcification of infancy. Dis. Model. Mech..

[134] Schafer C., Heiss A., Schwarz A., Westenfeld R., Ketteler M., Floege J., Muller-Esterl W., Schinke T., Jahnen-Dechent W. (2003). The serum protein alpha 2-Heremans-Schmid glycoprotein/fetuin-A is a systemically acting inhibitor of ectopic calcification. J. Clin. Investig..

[135] Jiang Q., Dibra F., Lee M.D., Oldenburg R., Uitto J. (2010). Overexpression of fetuin-a counteracts ectopic mineralization in a mouse model of pseudoxanthoma elasticum (abcc6 (−/−)). J. Investig. Dermatol..

[136] Huang J., Snook A.E., Uitto J., Li Q. (2019). Adenovirus-Mediated ABCC6 Gene Therapy for Heritable Ectopic Mineralization Disorders. J. Investig. Dermatol..

[137] La Russo J., Jiang Q., Li Q., Uitto J. (2008). Ectopic mineralization of connective tissue in Abcc6(−/−) mice: Effects of dietary modifications and a phosphate binder—A preliminary study. Exp. Dermatol..

[138] Gorgels T.G.M.F., Waarsing J.H., de Wolf A., Brink J.B.T., Loves W.J.P., Bergen A.A.B. (2010). Dietary magnesium, not calcium, prevents vascular calcification in a mouse model for pseudoxanthoma elasticum. J. Mol. Med..

[139] La Russo J., Li Q., Jiang Q., Uitto J. (2009). Elevated dietary magnesium prevents connective tissue mineralization in a mouse model of pseudoxanthoma elasticum (Abcc6 (−/−)). J. Investig. Dermatol..

[140] Gorgels T.G.M.F., Waarsing J.H., Herfs M., Versteeg D., Schoensiegel F., Sato T., Schlingemann R.O., Ivandic B., Vermeer C., Schurgers L.J. (2011). Vitamin K supplementation increases vitamin K tissue levels but fails to counteract ectopic calcification in a mouse model for pseudoxanthoma elasticum. J. Mol. Med..

[141] Jiang Q., Li Q., Grand-Pierre A.E., Schurgers L.J., Uitto J. (2011). Administration of vitamin K does not counteract the ectopic mineralization of connective tissues in Abcc6 (−/−) mice, a model for pseudoxanthoma elasticum. Cell Cycle.

[142] Carrillo-Linares J.L., García-Fernández M.I., Morillo M.J., Sánchez P., Rioja J., Barón F.J., Ariza M.J., Harrington D.J., Card D., Boraldi F. (2018). The Effects of Parenteral K1 Administration in Pseudoxanthoma Elasticum Patients Versus Controls. A Pilot Study. Front. Med..

[143] Nollet L., van Gils M., Verschuere S., Vanakker O. (2019). The Role of Vitamin K and Its Related Compounds in Mendelian and Acquired Ectopic Mineralization Disorders. Int. J. Mol. Sci..

[144] Bauer C., le Saux O., Pomozi V., Aherrahrou R., Kriesen R., Stölting S., Liebers A., Kessler T.S.H., Erdmann J., Aherrahrou Z. (2018). Etidronate prevents but does not completely reverse dystrophic cardiac calcification by.2 inhibiting macrophage aggregation. Sci. Rep..

[145] Otero J.E., Gottesman G.S., McAlister W.H., Mumm S., Madson K.L., Kiffer-Moreira T., Sheen C., Millán J.L., Ericson K.L., Whyte M.P. (2013). Severe skeletal toxicity from protracted etidronate therapy for generalized arterial calcification of infancy. J. Bone Miner. Res..

[146] Chong C.R., Hutchins G.M. (2008). Idiopathic infantile arterial calcification: The spectrum of clinical presentations. Pediatr. Dev. Pathol..

[147] Dabisch-Ruthe M., Kuzaj P., Götting C., Knabbe C., Hendig D. (2014). Pyrophosphates as a major inhibitor of matrix calcification in *Pseudoxanthoma elasticum*. J. Dermatol. Sci..

[148] Li Q., Kingman J., Sundberg J.P., Levine M.A., Uitto J. (2016). Etidronate prevents, but does not reverse, ectopic mineralization in a mouse model of pseudoxanthoma elasticum (Abcc6−/−). Oncotarget.

[149] Jacobs I.J., Li D., Ivarsson M.E., Uitto J., Li Q. (2021). A phytic acid analogue INS-3001 prevents ectopic calcification in an Abcc6(−/−) mouse model of pseudoxanthoma elasticum. Exp. Dermatol..

[150] Hosen M.J., van Nieuwerburgh F., Steyaert W., Deforce D., Martin L., Leftheriotis G., de Paepe A., Coucke P.J., Vanakker O.M. (2015). Efficiency of Exome Sequencing for the Molecular Diagnosis of Pseudoxanthoma Elasticum. J. Investig. Dermatol..

[151] Plomp A.S., Bergen A.A., Florijn R.J., Terry S.F., Toonstra J., van Dijk M.R., de Jong P.T. (2009). Pseudoxanthoma elasticum: Wide phenotypic variation in homozygotes and no signs in heterozygotes for the c.3775delT mutation in ABCC6. Genet. Med..

[152] Uitto J., Jiang Q., Varadi A., Bercovitch L.G., Terry S.F. (2014). Pseudoxanthoma Elasticum: Diagnostic Features, Classification, and Treatment Options. Expert Opin. Orphan Drugs.

[153] Bartstra J.W., Risseeuw S., de Jong P.A., van Os B., Kalsbeek L., Mol C., Baas A.F., Verschuere S., Vanakker O., Florijn R.J. (2021). Genotype-phenotype correlation in pseudoxanthoma elasticum. Atherosclerosis.

[154] Boraldi F., Costa S., Rabacchi C., Ciani M., Vanakker O., Quaglino D. (2014). Can APOE and MTHFR poly-morphisms have an influence on the severity of cardiovascular manifestations in Italian Pseudoxanthoma elasticum affected patients?. Mol. Genet. Metab. Rep..

[155] Hendig D., Knabbe C., Götting C. (2013). New insights into the pathogenesis of pseudoxanthoma elasticum and related soft tissue calcification disorders by identifying genetic interactions and modifiers. Front. Genet..

[156] Vanakker O.M.M., Hosen M.J., de Paepe A. (2013). The ABCC6 transporter: What lessons can be learnt from other ATP-binding cassette transporters?. Front. Genet..

[157] Navasiolava N., Gnanou M., Douillard M., Saulnier P., Aranyi T., Ebran J.-M., Henni S., Humeau H., Lefthériotis G., Martin L. (2019). The extent of pseudoxanthoma elasticum skin changes is related to cardiovascular complications and visual loss: A cross-sectional study. Br. J. Dermatol..

[158] Zhao J., Kingman J., Sundberg J.P., Uitto J., Li Q. (2017). Plasma PPi Deficiency Is the Major, but Not the Exclusive, Cause of Ectopic Mineralization in an Abcc6 Mouse Model of PXE. J. Investig. Dermatol..

[159] Mention P.J., Lacoeuille F., Leftheriotis G., Martin L., Omarjee L. (2018). 18F-Flurodeoxyglucose and 18F-Sodium Fluoride Positron Emission Tomography/Computed Tomography Imaging of Arterial and Cu-taneous Alterations in Pseudoxanthoma Elasticum. Circ. Cardiovasc. Imaging.

[160] Oudkerk S.F., de Jong P.A., Blomberg B.A., Scholtens A.M., Mali W.P., Spiering W. (2016). Whole-Body Visualization of Ectopic Bone Formation of Arteries and Skin in Pseudoxanthoma Elasticum. JACC Cardiovasc. Imaging.

[161] Humeau-Heurtier A., Colominas M.A., Schlotthauer G., Etienne M., Martin L., Abraham P. (2017). Bidimensional unconstrained optimization approach to EMD: An algorithm revealing skin perfusion alterations in pseudoxanthoma elasticum patients. Comput. Methods Programs Biomed..

[162] Gass J.D. (2003). “Comet” lesion: An ocular sign of pseudoxanthoma elasticum. Retina.

[163] Ciulla T.A., Rosenfeld P.J. (2009). Anti-vascular endothelial growth factor therapy for neovascular ocular diseases other than age-related macular degeneration. Curr. Opin. Ophthalmol..

[164] Vasseur M., Carsin-Nicol B., Ebran J., Willoteaux S., Martin L., Leftheriotis G. (2011). Carotid Rete Mirabile and Pseudoxanthoma Elasticum: An Accidental Association?. Eur. J. Vasc. Endovasc. Surg..

[165] Pavlovic A.M., Zidverc-Trajkovic J., Milovic M.M., Pavlovic D.M., Jovanovic Z., Mijajlovic M., Petrovic M., Kostic V.S., Sternic N. (2005). Cerebral Small Vessel Disease in Pseudoxanthoma Elasticum: Three Cases. Can. J. Neurol. Sci..

[166] Miglionico R., Ostuni A., Armentano M.F., Milella L., Crescenzi E., Carmosino M., Bisaccia F. (2017). ABCC6 knockdown in HepG2 cells induces a senescent-like cell phenotype. Cell. Mol. Biol. Lett..

[167] Tiemann J., Wagner T., Lindenkamp C., Plumers R., Faust I., Knabbe C., Hendig D. (2020). Linking ABCC6 Deficiency in Primary Human Dermal Fibroblasts of PXE Patients to p21-Mediated Premature Cellular Se-nescence and the Development of a Proinflammatory Secretory Phenotype. Int. J. Mol. Sci..

[168] Mungrue I.N., Zhao P., Yao Y., Meng H., Rau C., Havel J.V., Gorgels T.G.M.F., Bergen A.A., MacLellan W.R., Drake T.A. (2011). Abcc6 Deficiency Causes Increased Infarct Size and Apoptosis in a Mouse Cardiac Ischemia-Reperfusion Model. Arter. Thromb. Vasc. Biol..

[169] Hu X., Peek R., Plomp A., Brink J.T., Scheffer G., van Soest S., Leys A., de Jong P.T.V.M., Bergen A.A.B. (2003). Analysis of the Frequent R1141X Mutation in theABCC6Gene in Pseudoxanthoma Elasticum. Investig. Ophthalmol. Vis. Sci..

[170] Pfendner E.G., Uitto J., Gerard G.F., Terry S.F. (2008). Pseudoxanthoma elasticum: Genetic diagnostic markers. Expert Opin. Med. Diagn..

[171] Keeling K.M., Bedwell D.M. (2011). Suppression of nonsense mutations as a therapeutic approach to treat genetic diseases. Wiley Interdiscip. Rev. RNA.

[172] Wilschanski M., Miller L.L., Shoseyov D., Blau H., Rivlin J., Aviram M., Cohen M., Armoni S., Yaakov Y., Pugatch T. (2011). Chronic ataluren (PTC124) treatment of nonsense mutation cystic fibrosis. Eur. Respir. J..

[173] Kerem E.E., Konstan M.M., de Boeck K., Accurso F.F., Sermet-Gaudelus I., Wilschanski M.M., Elborn S.J., Melotti P.P., Bronsveld I.I., Fajac I. (2014). Ataluren for the treatment of nonsense-mutation cystic fibrosis: A randomised, double-blind, placebo-controlled phase 3 trial. Lancet Respir. Med..

[174] Dover G.J., Brusilow S., Samid D. (1992). Increased Fetal Hemoglobin in Patients Receiving Sodium 4-Phenylbutyrate. N. Engl. J. Med..

[175] Maestri N.E., Brusilow S.W., Clissold D.B., Bassett S.S. (1996). Long-Term Treatment of Girls with Ornithine Transcarbamylase Deficiency. N. Engl. J. Med..

[176] Perrine S.P., Ginder G.D., Faller D.V., Dover G.H., Ikuta T., Witkowska H.E., Cai S.P., Vichinsky E.P., Olivieri N.F. (1993). A short-term trial of butyrate to stimulate fetal-globin-gene expression in the beta-globin disorders. N. Engl. J. Med..

[177] McGuire B.M., Zupanets I.A., Lowe M.E., Xiao X., Syplyviy V.A., Monteleone J., Gargosky S., Dickinson K., Martinez A., Mokhtarani M. (2010). Pharmacology and safety of glycerol phenyl-butyrate in healthy adults and adults with cirrhosis. Hepatology.

[178] Iannitti T., Palmieri B. (2011). Clinical and experimental applications of sodium phenylbutyrate. Drugs R D.

[179] Gonzales E., Grosse B., Schuller B., Davit-Spraul A., Conti F., Guettier C., Cassio D., Jacquemin E. (2015). Targeted pharmacotherapy in progressive familial intrahepatic cholestasis type 2: Evidence for improvement of cholestasis with 4-phenylbutyrate. Hepatology.

[180] Hayashi H., Naoi S., Hirose Y., Matsuzaka Y., Tanikawa K., Igarashi K., Nagasaka H., Kage M., Inui A., Kusuhara H. (2016). Successful treatment with 4-phenylbutyrate in a patient with benign recurrent intrahepatic cholestasis type 2 refractory to biliary drainage and bilirubin absorption. Hepatol. Res..

[181] Rubenstein R.C., Zeitlin P.L. (2000). Sodium 4-phenylbutyrate downregulates Hsc70: Implications for intra-cellular trafficking of DeltaF508-CFTR. Am. J. Physiol. Cell. Physiol..

[182] Sorrenson B., Suetani R.J., Williams M.J.A., Bickley V.M., George P.M., Jones G.T., McCormick S.P.A. (2013). Functional rescue of mutant ABCA1 proteins by sodium 4-phenylbutyrate. J. Lipid Res..

[183] Millán J.L., Whyte M.P. (2016). Alkaline Phosphatase and Hypophosphatasia. Calcif. Tissue Int..

[184] Narisawa S., Harmey D., Yadav M.C., O’Neill W.C., Hoylaerts M.F., Millán J.L. (2007). Novel Inhibitors of Alkaline Phosphatase Suppress Vascular Smooth Muscle Cell Calcification. J. Bone Miner. Res..

[185] Sheen C.R., Kuss P., Narisawa S., Yadav M.C., Nigro J., Wang W., Chhea T.N., Sergienko E.A., Kapoor K., Jackson M.R. (2015). Pathophysiological Role of Vascular Smooth Muscle Alkaline Phosphatase in Medial Artery Calcification. J. Bone Miner. Res..

[186] Savinov A.Y., Salehi M., Yadav M.C., Radichev I., Millan J.L., Savinova O.V. (2015). Transgenic Overexpression of Tissue-Nonspecific Alkaline Phosphatase (TNAP) in Vascular Endothelium Results in Generalized Arterial Calcification. J. Am. Heart Assoc..

[187] St Hilaire C., Ziegler S.G., Markello T.C., Brusco A., Groden C., Gill F., Carlson-Donohoe H., Lederman R.J., Chen M.Y., Yang D. (2011). NT5E mutations and arterial calcifications. N. Engl. J. Med..

[188] Li Q., Price T.P., Sundberg J.P., Uitto J. (2014). Juxta-articular joint-capsule mineralization in CD73 deficient mice: Similarities to patients with NT5E mutations. Cell Cycle.

[189] Lee C.S., Bishop E.S., Zhang R., Yu X., Farina E.M., Yan S., Zhao C., Zheng Z., Shu Y., Wu X. (2017). Adenovirus-Mediated Gene Delivery: Potential Applications for Gene and Cell-Based Therapies in the New Era of Personalized Medicine. Genes Dis..

[190] De Baaij J.H.F., Hoenderop J.G.J., Bindels R.J.M. (2015). Magnesium in Man: Implications for Health and Disease. Physiol. Rev..

[191] Ter B.A.D., Shanahan C.M., de Baaij J.H.F. (2017). Magnesium Counteracts Vascular Calcification: Passive Interference or Active Modulation?. Arter. Thromb Vasc. Biol..

[192] Alfrey A.C., Miller N.L., Trow R. (1974). Effect of Age and Magnesium Depletion on Bone Magnesium Pools in Rats. J. Clin. Investig..

[193] Sakaguchi Y., Hamano T., Isaka Y. (2018). Magnesium and Progression of Chronic Kidney Disease: Benefits Beyond Cardiovascular Protection?. Adv. Chronic Kidney Dis..

[194] D’Marco L., Lima-Martínez M., Karohl C., Chacín M., Bermúdez V. (2020). Pseudoxanthoma Elasticum: An Interesting Model to Evaluate Chronic Kidney Disease-Like Vascular Damage without Renal Disease. Kidney Dis..

[195] Schinke T., McKee M.D., Karsenty G. (1999). Extracellular matrix calcification: Where is the action?. Nat. Genet..

[196] Gheduzzi D., Boraldi F., Annovi G., Devincenzi C.P., Schurgers L.J., Vermeer C., Quaglino D., Ronchetti I.P. (2007). Matrix Gla protein is involved in elastic fiber calcification in the dermis of pseudoxanthoma elasticum patients. Lab. Investig..

[197] Vermeer C. (1990). Gamma-carboxyglutamate-containing proteins and the vitamin K-dependent carboxylase. Biochem. J..

[198] Li Q., Jiang Q., Schurgers L.J., Uitto J. (2007). Pseudoxanthoma elasticum: Reduced gamma-glutamyl carboxylation of matrix gla protein in a mouse model (Abcc6−/−). Biochem. Biophys. Res. Commun..

[199] Vanakker O.M., Martin L., Gheduzzi D., Leroy B.P., Loeys B.L., Guerci V.I., Matthys D., Terry S.F., Coucke P.J., Pasquali-Ronchetti I. (2007). Pseudoxanthoma Elasticum-Like Phenotype with Cutis Laxa and Multiple Coagulation Factor Deficiency Represents a Separate Genetic Entity. J. Investig. Dermatol..

[200] Borst P., van de Wetering K., Schlingemann R. (2008). Does the absence of ABCC6 (multidrug resistance protein 6) in patients with Pseudoxanthoma elasticum prevent the liver from providing sufficient vitamin K to the periphery?. Cell Cycle.

[201] Vanakker O.M., Martin L., Schurgers L.J., Quaglino D., Costrop L., Vermeer C., Pasquali-Ronchetti I., Coucke P.J., de Paepe A. (2010). Low serum vitamin K in PXE results in defective carboxylation of mineralization inhibitors similar to the GGCX mutations in the PXE-like syndrome. Lab. Investig..

[202] Boraldi F., Annovi G., Guerra D., Devincenzi C.P., Garcia-Fernandez M.I., Panico F., de Santis G., Tiozzo R., Ronchetti I., Quaglino D. (2009). Fibroblast protein profile analysis highlights the role of oxidative stress and vitamin K recycling in the pathogenesis of pseudoxanthoma elasticum. Proteom. Clin. Appl..

[203] Huesa C., Staines K.A., Millan J.L., MacRae V.E. (2015). Effects of etidronate on the Enpp1(-)/(-) mouse model of generalized arterial calcification of infancy. Int. J. Mol. Med..

[204] Lomashvili K.A., Monier-Faugere M.C., Wang X., Malluche H.H., O’Neill W.C. (2009). Effect of bisphosphonates on vascular calcification and bone metabolism in experimental renal failure. Kidney Int..

[205] Oliveira J.R.M., Oliveira M.F. (2016). Primary brain calcification in patients undergoing treatment with the biphosphanate alendronate. Sci. Rep..

[206] Fleisch H., Bisaz S. (1962). Mechanism of Calcification: Inhibitory Role of Pyrophosphate. Nat. Cell Biol..

[207] O’Neill W.C., Lomashvili K.A., Malluche H.H., Faugere M.C., Riser B.L. (2011). Treatment with pyrophosphate inhibits uremic vascular calcification. Kidney Int..

[208] Orriss I.R., Arnett T.R., Russell R.G.G. (2016). Pyrophosphate: A key inhibitor of mineralisation. Curr. Opin. Pharmacol..

[209] Grases F., Sanchis P., Perello J., Isern B., Prieto R.M., Fernandez-Palomeque C., Fiol M., Bonnin O., Torres J.J. (2006). Phytate (Myo-inositol hexakisphosphate) inhibits cardiovascular calcifications in rats. Front. Biosci..

[210] Van den Berg C.J., Hill L.F., Stanbury S.W. (1972). Inositol phosphates and phytic acid as inhibitors of biological calcification in the rat. Clin. Sci..

[211] Schantl A.E., Verhulst A., Neven E., Behets G.J., D’Haese P.C., Maillard M., Mordasini D., Phan O., Burnier M., Spaggiari D. (2020). Inhibition of vascular calcification by inositol phosphates derivatized with ethylene glycol oligomers. Nat. Commun..

[212] Dennie W., McBride C. (1923). Treatment of arsphenamine dermatitis and certain other metallic poisonings. Arch. Dermatol. Syphilol..

[213] Halliday H.M., Sutherland C.E. (1925). Arsenical Poisoning Treated by Sodium Thiosulphate. BMJ.

[214] Hayden M.R., Goldsmith D.J.A. (2010). Sodium Thiosulfate: New Hope for the Treatment of Calciphylaxis. Semin. Dial..

[215] Arányi T., Bacquet C., de Boussac H., Ratajewski M., Pomozi V., Fülöp K., Brampton C.N., Pulaski L., le Saux O., Váradi A. (2013). Transcriptional regulation of the ABCC6 gene and the background of impaired function of missense disease-causing mutations. Front. Genet..

[216] Douet V., Heller M.B., le Saux O. (2007). DNA methylation and Sp1 binding determine the tissue-specific transcriptional activity of the mouse Abcc6 promoter. Biochem. Biophys. Res. Commun..

[217] Douet V., van Wart C.M., Heller M.B., Reinhard S., le Saux O. (2006). HNF4alpha and NF-E2 are key transcriptional regulators of the murine Abcc6 gene expression. Biochim. Biophys. Acta.

[218] Väärämäki S., Pelttari S., Uusitalo H., Tokesi N., Varadi A., Nevalainen P. (2019). Pyrophosphate Treatment in Pseudoxanthoma Elasticum (PXE)-Preventing ReOcclusion After Surgery for Critical Limb Ischaemia. Surg. Case Rep..

[219] Leopold J.A. (2013). Vascular calcification: An age-old problem of old age. Circulation.

[220] Rennenberg R.J.M.W., Kessels A.G.H., Schurgers L.J., Van Engelshoven J.M.A., de Leeuw P., Kroon A.A. (2009). Vascular calcifications as a marker of increased cardiovascular risk: A meta-analysis. Vasc. Health Risk Manag..

[221] Kranenburg G., Baas A.F., de Jong P.A., Asselbergs F.W., Visseren F.L.J., Spiering W. (2019). The prevalence of pseudoxanthoma elasticum: Revised estimations based on genotyping in a high vascular risk cohort. Eur. J. Med. Genet..

[222] Buchheiser A., Ebner A., Burghoff S., Ding Z., Romio M., Viethen C., Lindecke A., Köhrer K., Fischer J.W., Schrader J. (2011). Inactivation of CD73 promotes atherogenesis in apolipoprotein E-deficient mice. Cardiovasc. Res..

[223] Jalkanen J., Hollmén M., Jalkanen S., Hakovirta H. (2016). Regulation of CD73 in the development of lower limb atherosclerosis. Purinergic Signal..

[224] Nitschke Y., Weissen-Plenz G., Terkeltaub R., Rutsch F. (2011). Npp1 promotes atherosclerosis in ApoE knockout mice. J. Cell. Mol. Med..

[225] Sutton N.R., Bouïs D., Mann K.M., Rashid I.M., McCubbrey A.L., Hyman M.C., Goldstein D.R., Mei A., Pinsky D.J. (2020). CD73 Promotes Age-Dependent Accretion of Atherosclerosis. Arter. Thromb. Vasc. Biol..

[226] Li Q., Guo H., Chou D.W., Berndt A., Sundberg J.P., Uitto J. (2014). Mouse Models for Pseudoxanthoma Elasticum: Genetic and Dietary Modulation of the Ectopic Mineralization Phenotypes. PLoS ONE.

